# Happy-Productive Teams and Work Units: A Systematic Review of the ‘Happy-Productive Worker Thesis’

**DOI:** 10.3390/ijerph17010069

**Published:** 2019-12-20

**Authors:** M. Esther García-Buades, José M. Peiró, María Isabel Montañez-Juan, Malgorzata W. Kozusznik, Silvia Ortiz-Bonnín

**Affiliations:** 1 Department of Psychology, University of the Balearic Islands, 07122 Palma de Mallorca, Spain; m.montanez@uib.es (M.I.M.-J.); silvia.ortiz@uib.es (S.O.-B.); 2 IDOCAL (Institut d’Investigació en Psicologia del RRHH, del Desenvolupament Organitzacional i de la Qualitat de Vida Laboral), University of Valencia, 46010 Valencia, Spain; 3 IVIE (Instituto Valenciano de Investigaciones Económicas), 46020 Valencia, Spain; 4 Work, Organizational and Personnel Psychology Research Group (WOPP), KU Leuven, 3000 Leuven, Belgium; gosia.kozusznik@kuleuven.be

**Keywords:** happy, productive, performance, satisfaction, affect, engagement, team, work-unit

## Abstract

The happy-productive worker thesis (HPWT) assumes that happy employees perform better. Given the relevance of teams and work-units in organizations, our aim is to analyze the state of the art on happy-productive work-units (HPWU) through a systematic review and integrate existing research on different collective well-being constructs and collective performance. Research on HPWU (30 studies, 2001–2018) has developed through different constructs of well-being (hedonic: team satisfaction, group affect; and eudaimonic: team engagement) and diverse operationalizations of performance (self-rated team performance, leader-rated team performance, customers’ satisfaction, and objective indicators), thus creating a disintegrated body of knowledge about HPWU. The theoretical frameworks to explain the HPWU relationship are attitude–behavior models, broaden-and-build theory, and the job-demands-resources model. Research models include a variety of antecedents, mediators, and moderating third variables. Most studies are cross-sectional, all propose a causal happy–productive relationship (not the reverse), and generally find positive significant relationships. Scarce but interesting time-lagged evidence supports a causal chain in which collective well-being leads to team performance (organizational citizenship behavior or team creativity), which then leads to objective work-unit performance. To conclude, we identify common issues and challenges across the studies on HPWU, and set out an agenda for future research.

## 1. Introduction

The happy-productive worker thesis (HPWT) has a long tradition in work and organizational psychology since the human relations movement (Hawthorne studies in the 1930s). This movement showed the importance of groups in affecting the behavior of individuals at work and strongly contributed to the generalized belief that a happy worker is more productive. Years later, an influential review expanded the widespread belief that the relationship between satisfaction and job performance was just an ‘illusory correlation’ (r = 0.17) [1]. However, re-calculations of those results [2] and more recent meta-analyses highlighted the job attitudes–job performance relationship as a relevant topic worth further research and applied interest (r = 0.30) [3,4]. More recently, research on the relationship between well-being and performance has expanded to other constructs such as affect [5] and engagement [6,7]. Some scholars view the happy–performance relationship as weak, spurious, or questionable [2,8], and many consider well-being and performance as unrelated variables [2,9]. On the other hand, different meta-analyses have demonstrated a positive significant relationship between individual well-being and task performance [4,7]. 

Most research on HPWT has taken place at the individual level. However, the changes and transformation in the world of work and organizations has led to a growing relevance of work teams and work-units in current organizations. More than half of all employees in the 28 member states of the European Union work in a team that has common tasks and can plan its work [10]. Despite the importance of teams in organizational life, studies on the HPWT at the team and work-unit level is still scant. Moreover, research on this issue has often relied on single constructs of collective well-being such as ‘group affect’ [11] or ‘work-unit satisfaction’ [12]. Over the last decades, several quantitative studies have investigated happy-productive teams. Yet, to date, there has been no systematic review bringing together and synthesizing existing research on this topic. To fill this research gap, our aim is to analyze the state of the art on happy–productive work-units (HPWU) through a systematic review and integrate existing research on different collective well-being constructs and collective performance. A systematic review would provide a comprehensive picture on the current knowledge on HPWU, a better understanding of the strengths, commonalities and differences across constructs, and provide implications for team management and future research.

To achieve our main objective, we undertake a systematic review of peer-reviewed research on HPWU from 2001 to 2018. Considering the limitations of HPWT research at the individual level [9,13,14], we explore research on eudaimonic constructs of well-being/happiness as well as hedonic constructs, and consider multiple aspects of collective performance and sources of evaluation. Furthermore, we review the literature on HPWU by placing the focus on answering three research questions: (1) Which are the main features of the conceptualization and measurement of collective well-being? (2) Which theoretical frameworks are used to explain the collective HPWU relationship and which third variables are included in HPWU research models? (3) What is the evidence for causal or reciprocal relationships between collective wellbeing and collective performance?

In this review, we first describe the conceptualization of the two key constructs in the HPWT (happiness and productivity). Second, we explain the methodological approach adopted for the systematic review. In the results section, we present a brief description of the studies identified, and then proceed to report the main findings structured around the research questions. Finally, we discuss the state of the art of research on HPWU, limitations, and challenges for future research. 

### 1.1. Happiness and Well-Being at Work 

Scholars have treated happiness as well-being and have studied it through different constructs that overlap with the broad concept of happiness (e.g., psychological well-being, subjective well-being, satisfaction with life). There are two main perspectives about happiness or well-being: hedonic and eudaimonic [15]. The “hedonic approach focuses on happiness and defines well-being in terms of pleasure attainment and pain avoidance; and the eudaimonic approach focuses on meaning and self-realization and defines well-being in terms of the degree to which a person is fully functioning” [16] (p. 141). Following Sonnentag [17], well-being refers to a person’s hedonic experience of feeling good and to the eudaimonic experience of fulfilment and purpose.

So far, research on the HPWT has focused mainly on hedonic constructs (i.e., job satisfaction, affect, and emotions). However, the last decades have seen a growth on research on individual-level eudaimonic constructs such as engagement or flow [6], thriving at work [18,19,20], flourishing at work [21], meaning at work [22], and purpose in life or personal growth [23]. In her review about happiness at work, Fisher [15] identified some research on collective job satisfaction, group task satisfaction, group affective tone, group mood, unit-level engagement. We present the hedonic and eudaimonic perspectives on individual and collective wellbeing at work identified by Fisher in Table 1. In our review of HPWU, we aim to broaden the scope of research on collective well-being beyond a hedonic perspective by also exploring whether research on eudaimonic constructs has taken place at the team/work-unit level.

Recognizing the social dimension of work, well-being may be studied at the collective level as research on affective and emotional climates has shown [24]. Collective happiness or well-being refers to an emotional or affective climate that emerges in work-units and becomes a work context for employees affecting their work experience, behaviors, and performance [25]. Emotional or affective climates emerge in teams as a sharedness of affective reactions or emotional responses [25]. Teams develop shared climates through both top-down and bottom-up processes [26]. Top-down processes stem from team members sharing their work environment, team manager, most of their tasks, and from their exposure to similar job conditions. Shared affective climates also emerge from bottom up processes including social interactions and communication, emotional contagion, role modelling, and advice giving [26]. 

Measuring collective well-being presents important methodological issues. Most measures of collective well-being arise from evaluations provided by individuals (i.e., team members), which are statistically aggregated to the collective level. A majority of researchers interested in group or team processes have adopted either a direct consensus or a referent-shift consensus model to aggregate individual responses [27,28]. In the direct consensus model, team members evaluate their individual well-being with items using an ‘individual referent’ (e.g., “I am enthusiastic about my job”). Referent-shift models require individual team members to respond to survey items, which refer directly to the team (e.g., “My team is enthusiastic about the task”). Items worded with a ‘team referent’ shift the respondents’ attention to the team level. The second step in both direct consensus and referent-shift models is to average individual responses to obtain a group-level measure (e.g., group’s statistical mean) after assuming and testing for some minimal level of within-group interrater agreement (IRA) and interrater reliability (IRR) consensus [29,30]. 

### 1.2. Collective Performance 

The approach taken to define and measure performance differs depending on the level at which performance is assessed (i.e., individual, team/work-unit, or organizational). At the team level, it is important to make a conceptual distinction between team performance and team effectiveness. Following Salas et al. [31]: 

“Team performance accounts for the outcomes of the team’s actions regardless of how the team may have accomplished the task. Conversely, team effectiveness takes a more holistic perspective in considering not only whether the team performed (e.g., completed the team task) but also how the team interacted (i.e., team processes, teamwork) to achieve the team outcome. This is an important differentiation because many factors external to the team may contribute to the success (or failure) of the team, and therefore in some cases team performance measures may be deficient in understanding the team” (p. 557).

**Figure 1:**
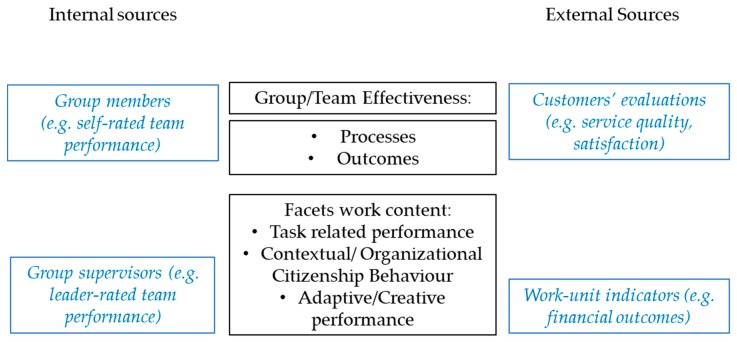
Work-unit effectiveness: facets and sources of evaluation.

Although team effectiveness is the appropriate term, to keep in line with the expressions used in the HPWT literature we will also use the expressions ‘collective performance’ and ‘productive teams’ to refer to measures of both the team’s achievements and actions for the remainder of the article. Building on previous research, we contend that a comprehensive evaluation of a team’s effectiveness needs to include measures of different aspects of the team’s interaction (processes) and performance (outcomes) [31], as well as different facets of the work content (e.g., task, organizational citizenship behavior (OCB), creativity) [9]; and multiple sources of evaluation (group members, supervisors, customers, and objective data) [32]. Figure 1 reflects these core aspects of work-unit effectiveness. Based on these aspects, we proceed to describe categories of collective performance commonly used in research [12,33]: team performance, customers’ evaluations, and work-unit objective/financial indicators. 

Team performance may refer to different aspects of the work content (e.g., task performance, contextual performance, and creativity performance) [9]; may refer to team members’ outcomes (i.e., do the team members achieve their objectives?) or processes (i.e., what do team members do when at work?); and may be provided by different agents, the team-members themselves (self-rated performance) or their supervisors. Typically, group/team members provide subjective ratings on their effectiveness based on their own perceptions (i.e., self-rated team performance). Team leaders (managers or supervisors) are also frequent evaluators of the team’s performance (i.e., leader-rated team performance). Managers’ evaluations of their work-unit’s performance are widespread and taken for valid as they are in the position to observe their team’s work and give a global evaluation of how much or how well the team works and accomplishes the set objectives. Managers typically provide a global measure about the work-unit. In our review, we call this measure of team’s effectiveness team performance and we will distinguish between self-rated team performance and leader-rated team performance, and whenever possible we will specify whether task performance, OCB, or creative performance are taken into account. 

Customers’ evaluations constitute another relevant source to assess team’s effectiveness. It is externally rated, and it usually reflects a combined evaluation of both processes and outcomes (i.e., how fast a team responds to customers’ requests or to which extent the solution to a problem is satisfactory). Customers’ evaluations typically include facets such as service quality and customer satisfaction. 

Another performance category includes work-unit objective/financial indicators. In this case, team productivity refers to a combination of efficiency and effectiveness and encompasses a number of results-oriented outcomes such as profit, return-on-investment, and sales [12]. These objective assessments of performance are usually recorded for groups rather than for individuals [14] and therefore refer to the work-unit as a whole.

The association between well-being and performance may vary with the type of performance considered [34]. The diversity in operationalizations of team performance provides a rich combination of criteria, thus increasing the interest in the evaluation of how collective well-being relates to different collective performance criteria. Overall, a more comprehensive consideration of collective well-being and collective performance allows for a richer picture of HPWU relationships. Therefore, in our systematic review, we aim to explore HPWU research considering both hedonic and eudaimonic constructs of well-being, and performance indicators based on multiple aspects of collective performance and multiple sources of evaluation. 

## 2. Materials and Method: Study Search and Collection

To address our research questions, we conducted a systematic literature review searching the PsycINFO and PsycARTICLES databases for empirical studies in peer-reviewed journal articles that addressed the HPWT in groups/teams/work-units between 2001 and 2018 published in English or Spanish. This search took place in June 2019. For a comprehensive inclusion of all potential terms referring to happy teams and productive teams, we used the following keywords (and combinations thereof): happy (well-being, satisfaction, affect, emotions, mood, engagement, flourishing, flow, purpose, meaning, hedonic, eudaimonic, morale); productive (performance, productivity, efficiency, effectiveness, customer satisfaction, OCB, innovation, creativity); and team (work-unit, work group). 

We included all studies about groups, teams, work-units, and branches because all represent the same meso-level of analysis as opposed to individual and organizational levels. We broadly define team or work-unit as a group of three or more employees who meet on a regular basis, are jointly responsible for one or more tasks, and are nested in a larger social system (e.g., organization) [35]. In this vein, we use the terms group and team interchangeably as is common in organizational psychology literature [32]. Although we recognize that some differences may exist, we focus on their communalities [33]. This exploratory systematic search yielded 356 abstracts. A first screening of all abstracts showed research to concentrate on three collective well-being constructs: satisfaction, group affect (emotions and mood), and engagement. We did not find studies analyzing eudaimonic constructs at the team level (e.g., meaning of work or flourishing). Consequently, we conducted three specific searches on satisfaction, group affect, and engagement, which we complemented with cross-references found in different meta-analyses and through a snowball system. The entire search phrases are presented in Supplementary Materials.

In each case, two independent evaluators analyzed all abstracts to check if they met two inclusion criteria: (1) the study reported collective level measurements of well-being and performance; (2) it presented empirical research undertaken with work samples (e.g. we excluded students and athletes). Agreement between evaluators reached 96%. After solving discrepancies, evaluators selected 87 abstracts.

In the next stage, we proceeded with full-text analysis. We searched and found 87 manuscripts. We discarded the studies that while studying team phenomena, analyzed the data at the individual or organizational level, or did not report correlations between well-being and performance. We also discarded nine studies on collective satisfaction and one on group affect, which did not propose a happy-productive or the reversed productive-happy research model. These 10 studies presented models akin to input-processes-outcomes models of team effectiveness and considered both collective well-being and collective performance as dependent variables. 

**Figure 2:**
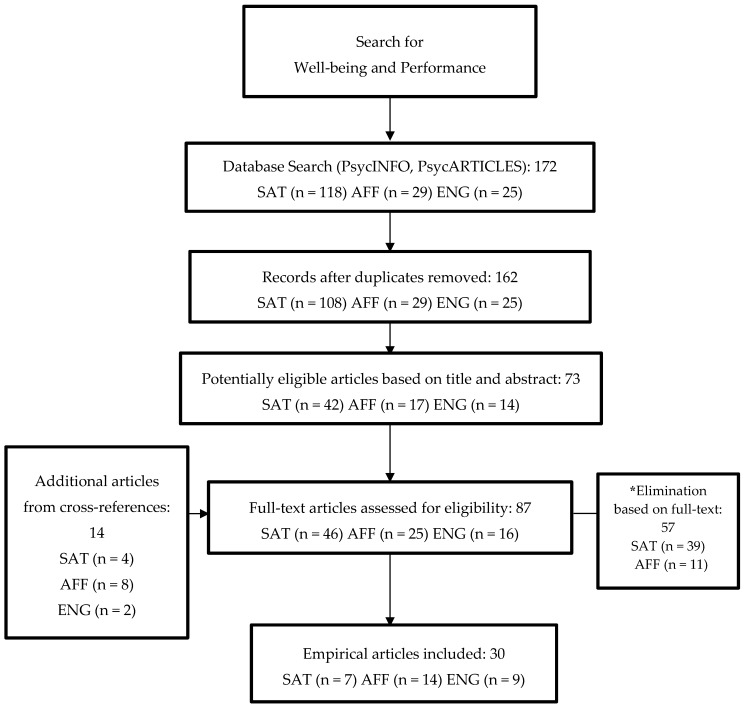
Process of analysis and selection of research papers on happy–productive teams and work-units. Notes. SAT = satisfaction; AFF = group affect; ENG = engagement; *Exclusion criteria: sample (no work sample), quality of the study (meta-analyses, review), analyses (individual data analyses, no correlation data), measures (no satisfaction measures, no performance measures), and happy–productive relationship (happy and productive as dependent variables).

The final sample of empirical studies with this systematic literature review yielded 30 studies relating happy work-units and performance strictly at the collective level of analysis. A PRISMA (Preferred Reporting Items for Systematic reviews and Meta-Analyses) flowchart (Figure 2) summarizes the process of search, analysis, and selection of research papers.

### Data Analysis

First, we read the 30 manuscripts and extracted relevant information which we report in the Appendix A (Table A1 for satisfaction, Table A2 for group affect, and Table A3 for team engagement) about their study goal, theoretical background, direction proposed between happiness and productivity (HP: happy–productive; PH: productive–happy), definition and operationalization of collective well-being and performance, study design (cross-sectional or time-lagged), reported correlations, and sample. Next, we proceeded to analyze the manuscripts in order to answer our main research questions and summarize the findings in the results sections.

## 3. Results

### 3.1. Description of the Studies

#### 3.1.1. Collective Satisfaction

We identified seven empirical studies relating collective satisfaction and collective performance in work-units or teams. Samples were drawn from different sectors (sales, manufacturing, social care, local governments, health care, banks), and countries (USA, Netherlands, United Kingdom, Taiwan, China, and Australia) with representations from four continents. Samples sizes ranged from 28 to 171 work-units. Most studies used a cross-sectional design, with three using time-lagged performance indicators [33,36,37].

#### 3.1.2. Group Affect

We identified 14 empirical studies relating group affect and collective performance in work-units or teams. Samples were drawn from sectors such as electronic industry, service organizations, sales, banks, orchestras, etc., and different countries (Germany, Spain, Brazil, Taiwan, South Korea) with representations from three continents. Samples sizes ranged from 22 to 417 work-units. Most studies used a cross-sectional design, with two using time-lagged performance indicators [38,39].

#### 3.1.3. Team Engagement

We identified nine empirical studies relating team engagement and collective performance. Empirical research exploring the collective engagement-performance relationship varies considerably in terms of sample size (54 to 242 teams), types of company/sector (health services, hospitality, call centres, research teams, and teachers). All studies have taken place in European countries (Spain, Finland, UK, and The Netherlands) and one in Vietnam. All studies used a cross-sectional design.

### 3.2. Main Findings

#### 3.2.1. Research Question 1. Which Are the Main Features of the Conceptualization and Operationalization of Collective Well-Being?

In this section, we review the main features of the conceptualization and operationalization of collective well-being. We review the definitions, instruments, informants and referents used within the literature identified in the systematic review.

**Definition of Collective Satisfaction.** An important theoretical contribution in defining satisfaction at the unit-level as a different phenomenon to individual job satisfaction is the work by Whitman et al. [12] (p. 46). They defined “unit-level job satisfaction” as “a work unit’s shared internal state that is expressed by affectively and cognitively evaluating shared job experiences with some degree of favour or disfavour”. They stressed the relevance of *sharedness* as a critical precondition to forming collective job satisfaction. The antecedents to this sharedness are both situational (e.g., similar work environments and conditions) and dispositional (i.e., processes of attraction–selection–attrition). These antecedents lead to a common interpretation, understanding, and attitudinal evaluation of the job experience [12].

Within the reviewed literature, four studies omit a definition of collective satisfaction and three studies adopt the ”group task satisfaction” definition, which refers to “the group’s shared attitude toward its tasks and work environment” [12] (p. 1). Mason & Griffin [35] differentiate ”group task satisfaction” from ”individual job satisfaction” in that group-level attitudes will focus on the task for which the whole group is responsible and common aspects of the work environment rather than developing a shared attitude toward any one individual’s job. 

**Operationalization of Collective Satisfaction.** Operationalizations of collective satisfaction appear in diverse formats across studies: global vs. facets satisfaction, individual vs. team referents, and different instruments.

**Global vs. Facets Satisfaction.** Six studies reported global measures of job satisfaction, in which a few items capture an overall feeling about satisfaction with the team or work-unit (e.g., ”we are satisfied with each other’s contribution to our team”). Global satisfaction refers to a general attitude towards the team, and is distinct from satisfaction with facets or various features of the job. One study reported measures of satisfaction with facets such as tasks, rewards, supervision [35]. Both global and facets satisfaction scales are valid measures and preference for either one of them depends on the diagnostic vs. general purpose of the evaluation [40,41]. 

**Informants and Referents.** Team or work-unit members were the informants of job satisfaction in all studies and their responses were aggregated at the unit level. Six studies reported the use of individual referents (i.e., I am satisfied with…), and Mason & Griffin [35] used both individual and team referents (i.e., My team is satisfied with…) to measure aggregated “individual task satisfaction” and “group task performance” respectively. Regarding the debate about using individual or group referenced measures, Mason & Griffin [35] advocate the use of group referenced measures in preference to the individual referenced measures. In their empirical study, the group referenced ”group task satisfaction” measure explained variance in sportsmanship behavior and group absenteeism norms beyond aggregated “group members’ individual job satisfaction ratings”. Whitman et al. [12] in their meta-analysis compared the use of organization vs. job referent, finding unit-level organizational satisfaction more strongly related to unit-level performance (rho = 0.39) than unit-level job satisfaction (rho = 0.33). These results, although restricted to a limited amount of studies, suggest that the referent used does affect the satisfaction–performance relationship and the authors advocate the use of collective referents. 

**Collective Satisfaction Measures.** We found two validated instruments of satisfaction used at the collective level to grasp the extent to which members are satisfied with their teamwork. The “group task satisfaction scale” [35] consists of 10 items to tap into three dimensions: satisfaction with the task itself (e.g., work stimulating, fulfilling), satisfaction with the group’s internal work environment (e.g., the way they work together, conflict among team members), and satisfaction with the group’s external work environment (e.g., senior managers, support, resources, policies, rewards). This scale uses a group referent, i.e., “our team finds its work stimulating”. Furthermore, one study reported using the Minnesota Satisfaction Questionnaire [42] (20 items) to measure “aggregate individual job satisfactions” [35], and each one of the remaining studies used a different scale to the rest (two used 2-item scales, two used 3-item scales, one a 4-item scale, and one a 10-item scale).

**Summary Collective Satisfaction.** Group or team satisfaction has been defined as “a shared positive attitude towards a work-related object (i.e., the job, the team’s task, and the team’s environment)”. However, many studies have used individual referents and relied on a measure of “aggregated individual job satisfactions”. As an attitude, definitions incorporate both cognitive and affective evaluations of shared job experiences, but the evaluations of work-unit satisfaction are predominantly cognitive and stable [43] and “the affective property of job attitudes lay relatively inert” [43] (p. 362).

Overall, the lack of homogeneity in the use of instruments, number and content of items, and scale origin is remarkable. The widespread heterogeneity in operationalizations of team satisfaction is likely to affect the comparability of studies and results. We believe using validated team satisfaction scales (e.g., “group task satisfaction”), and combining global and team-facets satisfaction measures would strongly contribute to a more appropriate operationalization and understanding of team satisfaction and of its connection with team performance.

**Definition of Group Affect.** Group affect refers to the homogeneous affective states within the group [44] (p. 781). More specifically, it relates to the mood states team members experience or feel while on the job or in team meetings [45]. Research on group affect involves the study of affect, moods, and emotions at a collective level [5]. Most authors provide definitions of group positive affect with two components: “shared or homogeneous or consistent” and “affective states, feelings, affective reactions, emotions or moods”. They use the terms within each component as almost synonymous in their definitions of group affect, notwithstanding recognition of some differences among concepts (for instance, between emotions and moods) [46]. All these terms refer to how people feel, whether positive or negative (i.e., valence), and more or less activated (i.e., activation) [46].

Group affect, as a collectively shared pattern of affective states among group members, is a meaningful construct at the team level of analysis and an important factor that shapes group processes and outcomes [5]. Following Barsade & Gibson [47], group affect can be characterized through two approaches. A top-down approach in which group affect as a whole acts upon the emotions of the individuals within it, and a bottom-up approach in which group affect emerges as the result of the aggregate of individual group members’ affective states and traits. The group affect literature reviewed emphasizes the bottom-up approach and emotional contagion as the main mechanism explaining the emergence of group affect as a group level phenomenon. This view is complemented with the top-down influence of transformational leadership, which appears as a relevant antecedent of group affect within this literature. 

**Operationalization of Group Affect.** Seven studies used validated measures of positive group affect, namely six studies used PANAS (Positive and Negative Affective Scale) [48], and one used the Affective Well-being Scale [49]. The rest used a variety of scales ranging from 3–10 moods or emotions. PANAS has been criticized for its focus on high activation moods, and some researchers advocate complementing it with low activation moods [46]. 

The periods and statements accompanying the items are also diverse. One study measured group mood felt “at the very particular moment”, four studies have referred to “the past week”, one “in the last weeks”, one “in the past two weeks”, one “in the past six months”, two “in the past year”, two “in general”, and two do not specify time frames. There is a debate about the advantages and disadvantages of different time frames to measure group affect. Although “current mood states may be more accurately and reliably reported than recalled moods” [46] (p. 345), other authors suggest that group mood or emotion is a group’s temporally stable, basic temperament, with an overall positive or negative cast [50]. 

Additionally, operationalizations refer to how the team members have felt at work/job/at the store (five studies), at team meetings (three studies), or do not refer a particular situation (six studies). Regarding informants, team-members reported their positive affect in 13 studies, and only Rego et al. [38] had the store manager as an informant of team-members’ positive affect. They argue that “the store supervisor is, to a certain degree, an observer of the stores’ affective tone and behaves toward the store according to this perception/observation” (p. 69). 

**Summary Group Affect.** Group positive affect refers to how the team members have felt for a certain period of time (i.e., past week, or during a team meeting). Similar to collective satisfaction, group affect focuses on the affective component of working in a team or work-unit. As opposed to team satisfaction (with affective-cognitive components), there only appears an affective component, and there is no reference to specific aspects of the job/work, just affect (e.g., such as pleasure) while working or at work or at team meetings. 

**Definition of Team Work Engagement.** The concept of personal engagement was introduced by Kahn [51] as “the behaviors by which people … employ and express themselves physically, cognitively, and emotionally during role performances” (p. 694). Macey & Schneider [52] built on Kahn’s view to develop a theoretical framework that describes how some distal antecedents (i.e., job characteristics or leadership) influence engagement levels, which in turn affect performance outcomes. Furthermore, Schneider et al. [53] defined engagement as having two major components: the feelings of engagement or the heightened state of energy and enthusiasm associated with work and the organization, and engagement behaviors such as persistence of tasks, being proactive and taking on responsibilities when the need arises, all in the service of accomplishing organizational goals. This conceptualization of engagement has been applied to the organizational level [53,54] but to our knowledge not to the team level.

A second conceptualization with a business engagement perspective refers to engagement as “the individual’s involvement and satisfaction with as well as enthusiasm for work” (The Gallup Organization). This definition has been criticized for evaluating satisfaction together with, or instead of, engagement [52]; and the associated instrument (Gallup Q12, or Gallup Workplace Audit) for lacking face or construct validity [6,12]. Still, a meta-analysis with Q12 found a true score correlation of r = 0.42 between collective “satisfaction-engagement” and composite business-unit performance outcomes (e.g., customer satisfaction, productivity, profit, employee turnover, and accidents) in American for-profit companies [55]. A third stream of engagement research developed in Europe has become dominant [6] to a great extent due to the development of the Utrecht Work Engagement Scale (UWES) [56]. Within this perspective, engagement has been examined as a team-level construct [57]. 

Team engagement refers to “a positive, fulfilling, work-related and shared psychological state characterized by team work vigor, dedication and absorption which emerges from the interaction and shared experiences of the members of a work team” [58] (p. 107). Thus, engaged team members have high levels of energy and work hard (vigor), are enthusiastic about their work (dedicated), and are often fully immersed (absorbed) in their job so that time flies [59]. Emergence of team work engagement is attributed to the interaction and shared experiences of team members through two types of processes: implicit (i.e., emotional contagion) and explicit (i.e., team members sharing workplace experiences) [60]. A second definition of team engagement refers to it as “a shared, positive and fulfilling, motivational emergent state of work-related well-being” [61] (p. 35). Although Costa et al. [61] referred to engagement as a motivational state in their definition, they also contend that “Team work engagement seems to be a promising construct for future research on the affective and motivational emergent states of work teams” (p. 43).

Sonnentag [17] attempts to clarify conceptual boundaries and reflects on whether work engagement is a motivation or a well-being construct; she concludes that “work engagement and thriving as positive well-being concepts seem to be closely related to motivational and behavioral processes. Conceptually, however, they emphasize the experience of energy, dedication, absorption, and growth—as opposed to actual behaviors” (p. 264).

**Operationalization of Team Work Engagement**. We identified two measures of team work engagement: UWES and Team Work Engagement construct. In all cases, team members were the informants about the team’s work engagement and their responses were aggregated at the team level. Seven studies measured team engagement through different versions of the UWES scale: one used the 18-item version, one used the 17-item version, four used the 9-item version, and two used the 3-item version. Five studies used a team referent (i.e., “My team…”), three used an individual referent (i.e., “I am enthusiastic about my job”), and one study used both individual and team referents [62]. A second instrument, the team work engagement construct [61], has been validated to measure team work engagement and differentiate it from individual work engagement. It consists of nine items, measuring it as a team property with a team referent (i.e., “we are proud of the work we do”). Results show the nine items to converge in a single-factor structure. Two studies from our literature search used this instrument (4 and 9 items). 

**Summary Team Work Engagement.** Although different conceptualizations of engagement exist, when it comes to research of collective work engagement at the team level within our literature review, all authors have defined it following the Utrecht perspective: “a positive, fulfilling, work-related and shared psychological state characterized by team work vigor, dedication and absorption which emerges from the interaction and shared experiences of the members of a work team” [63]. Some authors have distinguished work engagement from job satisfaction in aspects such as level of activation (engagement high activation vs. satisfaction low activation), and work engagement from motivation [17]. Team work engagement is more related to an eudaimonic perspective of well-being, i.e., closer to feeling authentic and meaningful in one’s life [17], than a hedonic perspective emphasizing pleasure and absence of pain. Feeling engaged may be accompanied by positive and/or negative emotions. Thus, the main emotion in engagement is not pleasure like in hedonic constructs, but interest in order to pursue gratification [64]. Thus, engagement would explain team efforts in unpleasant conditions such as when team members ignore physical or mental exhaustion and continue working to achieve their objective. 

Unfortunately, operationalizations of collective engagement following the North American perspective based on the work by Kahn, and Macey and Schneider [52] have been applied to the organizational level [54,65] and to our knowledge not to the team level. All studies within our systematic review have followed the European perspective on work engagement and used the two validated measures of the construct at the team level: UWES and Team Work Engagement scale. These scales offer the benefits of consisting of a manageable amount of items, using team referents, offering adequate psychometric properties, and allowing for comparability among studies.

#### 3.2.2. Research Question 2: Which Theoretical Frameworks Are Used to Explain the Collective Happy-Productive Work-Unit Thesis? Which Third Variables Affect the Relationship between Well-Being and Performance in the Empirical Research Models (Mediators, Moderators, Antecedents)?

In this section, we describe the main theoretical frameworks underpinning the relationship between collective well-being and collective performance (see Table 2). We structure the findings around each of the collective well-being constructs. The relationship between collective well-being and performance is usually embedded in wider research models including third variables. Depending on the theoretical models and specific hypotheses, third variables may have a role as antecedents of the main variables, mediators between well-being and performance, or moderators that explain when or how the main HP relationship is stronger or weaker. Thus, we also describe third variables found in the research models.

**The Collective Satisfaction Literature: Theoretical Frameworks.** The seven studies identified in the systematic review considered collective satisfaction as an antecedent of collective performance. The main theoretical framework supporting the research models is the HPWT applied to the team level. In the early 1990s, Ostroff [66] applied the happy–productive thesis to the collective (organizational) level. She argued that satisfaction and the happiness of personnel would heighten organizational effectiveness through employees’ behaviors and responses at work. Building on the arguments from the sociotechnical and human relations schools, she proposed that positive attitudes trigger productivity-related behaviors, which in turn lead to organizational effectiveness. These productivity-related behaviors relevant to organizational effectiveness encompass attachment behaviors (i.e., attending to and staying in the organization), performance behaviors (i.e., job-related tasks) and citizenship behaviors (cooperation and collaborative efforts) [67]. A central mechanism is collaborative effort, in her words “satisfied employees will be more likely to engage in collaborative effort and accept organizational goals that can increase productivity, whereas dissatisfied employees … may fail to work collaboratively (p. 964)”. 

At the unit-level, Koys [33] proposed that “shared values or attitudes” are the key to the relationship between unit-level employee job satisfaction and organizational effectiveness. These shared attitudes lead to appropriate behaviors, which lead to organizational effectiveness. He also referred to collaboration as a key process between shared attitudes and productivity: “If a unit’s employees share positive attitudes, they should have norms of cooperation and collaboration, which in turn enhance unit productivity (p. 102)”. These first studies suggest the general idea that a shared attitude leads to collaborative behaviors among team members and subsequent improved work-unit performance. Following a similar reasoning, Whitman [12] proposed that OCB (e.g., a measure of team contextual performance) mediated the effects of work-unit satisfaction on performance (a composite of three criteria—productivity, withdrawal, and customer satisfaction). Testing this mediation through meta-analytical correlations, they found a small but significant mediator effect of OCB between satisfaction and performance. 

One study within the reviewed literature [36] found empirical support for a partial mediation of OCB between high performance work systems and departmental performance (e.g., overall departmental performance score based upon the percentage of success on each of the performance metrics tracked by the Welsh government) in a sample of 119 local government departments.

A related and complementary argument why happy work-units would be productive work-units refers to social exchange theory [68]. Thus, three studies propose that when (work-unit) employees are satisfied with their job or work-unit [33,69] or with high performance work systems provided by their companies [36], they would reciprocate with positive behaviors such as OCB to benefit the unit or organization. An additional theoretical background is applied to explaining the relationship between collective satisfaction and a specific type of performance (i.e., customer satisfaction). Three studies [33,37,70] refer to the service climate framework or the linkage research model [71,72] and the service-profit-chain model [73]. The service climate framework posits that a unit’s service climate (positive and strong-shared perception of service as a focus) leads to service behaviors, such as in-role behavior and customer-focused OCB as a mediator, which subsequently leads to positive customer experiences (quality, satisfaction, and loyalty). The service-profit-chain model posits that employees’ capability, satisfaction, and loyalty, would lead to satisfied and loyal customers, who tend to purchase more and increase organizational revenue and profits [33]. Two studies report positive and significant correlations between collective satisfaction and customer satisfaction [33,70]. 

Regarding empirical support, 15 out 22 correlations reported in cross-sectional studies proposing a happy–productive relationship are statistically significant and positive (range 0.17 to 0.63); seven with team task performance (range 0.27 to 0.63), three with team contextual performance (range 0.36 to 0.61), two with customer satisfaction (range 0.49 to 0.57) and four with objective financial criteria (range 0.19 to 0.43). Non-significant results are obtained between collective satisfaction and one measure of supervisor-rated performance [35], one with customer satisfaction [33], and three measures of financial profit [33,37]. 

**Collective Satisfaction: Third Variables Included in HP Research Models**. Antecedents affecting work-unit satisfaction (and collective performance) are related to transformational leadership and leader empowering behaviors, team task characteristics, high-performance work systems, and work-unit climate. In one study, leaders’ positive moods led to both transformational leadership and positive group affective tone, which then led to team processes such as team satisfaction, and in turn enhanced team sales performance [45]. A second study in a restaurant chain found that leader empowering behaviors increased work-units’ employees psychological empowerment, which in turn enhanced work-unit employee satisfaction, which consequently improved customer satisfaction [70]. Team task characteristics (task autonomy and feedback) were relevant antecedents of team member satisfaction, which together with task meaningfulness enhanced team performance [69]. In another study, high-performance work systems (HPWS) was an antecedent of departmental job satisfaction, which subsequently improved department performance [36]. Finally, Van De Voorde et al. [37] found that two work-unit climates (service orientation and goals orientation) increased work satisfaction.

Furthermore, Whitman et al. [12] analyzed in their meta-analysis the moderating role of several variables that need to be taken into account to understand when and under which conditions collective satisfaction and collective performance are related. Results showed that the satisfaction-performance relationship was moderated by the strength of unit consensus (rho = 0.32 for high consensus vs. rho = 0.22 for low consensus), industry type (stronger in the education vs. business sector); stronger for government units vs. for-profit sector. They concluded that “the strength of the relationship - though always positive-depended a great deal on how criteria were conceptualized, aligned, and constructed” (p. 72). In a meta-analysis on situational strength as a moderator of the relationship between job satisfaction and job performance [3], satisfied employees were more likely to be productive employees in those situations in which employees have a fair amount of discretion in deciding how to perform their work. We did not find similar studies at the work-unit level, but a similar moderator effect of discretionary behavior may exist for groups and work-units.

In summary, the theoretical arguments within the collective satisfaction literature refer to the HPWT, the general attitude-behavior link, social exchange theory, and linkage research model or service-profit chain. Both theory and empirical evidence suggests that contextual performance (e.g., OCB) is a mediator between collective satisfaction and objective performance, i.e., attitudes lead to collaborative behaviors. The HPWU relationship seems stronger for higher degrees of sharedness of collective satisfaction [12]. Work-unit satisfaction may be increased by antecedents such as leadership behaviors, team task design, HPWS, and work-unit climate.

**Group Affect Literature: Theoretical Frameworks.** All fourteen studies on group affect consider group affect as the antecedent of performance. The dominant theoretical framework in the group affect–performance literature is the “broaden-and-build” theory [74], which has been applied both at the individual and team level. This model has been complemented with the mood-as-input theory [75], recently applied to the team level. The rationale behind the broaden-and-build theory is that in a isomorphic way as it happens at the individual level, “positive group emotions may broaden the group’s range of attention, cognition, and action and build social resources such as friendship among the members” [76] (p. 74). In addition, positive emotions build long-term physical resources (e.g., better health), intellectual resources (e.g., knowledge), social resources (e.g., help and cooperation), and psychological resources (e.g., resilience) among individuals and team-members. 

Rhee [76] explored the role of group-member interactions as the underlying mechanism of the relationship between group emotions and group outcomes. She proposed three main interaction processes as mediators between positive group emotions and group outcomes: building on each other’s ideas, morale-building communication, and affirmation. Building on each other’s ideas among the group members (e.g., being attentive to others’ ideas and expanding the original idea to improve idea quality) is the manifestation of cognitive broadening and social spontaneity at the group level. On the other hand, morale-building (e.g., encouraging the group’s achievements and successful outcomes) and affirmation of each other’s ideas (e.g., accepting and supporting others’ opinions) manifest social spontaneity and building social resources. Rhee contends that these positive interaction processes have an effect on outcomes such as creativity, team-member learning, satisfaction with the group, and the quality of group decision making. 

The literature reviewed on group affect has proposed and tested cognitive, motivational, attitudinal, and behavioral team processes as mediators between group affective tone and team performance. For example, transformational leadership and positive affective tone enhanced team performance (perceived and objective) through team goal commitment (i.e., motivated team members pursue team goals), team satisfaction (i.e., satisfied members in terms of their team tasks and environments), and team helping behavior (i.e., team members exhibit more helping behaviors) in a sample of 85 sales teams in Taiwan [45]. In another study, positive emotions were positively related to team resilience (i.e., the process to face off, persevere and respond positively in the face of adversity), and team resilience was positively related to team in-role (i.e., task) and extra-role performance as reported by the supervisor in a sample of 216 teams [77]. 

Seong & Choi [78] reported a significant role of group positive affect in predicting group performance through group-level fit (i.e., the presence of shared goals among members and the collective pursuit of congruent goals) and group conflict in a sample of 96 Korean teams in the defence industry. Another study found support for the mediating role of team reflexivity (i.e., the extent to which team members collectively reflect on and communicate about the team’s objectives, strategies, and processes) and team promotion focus (i.e., team level motivational state that regulates and coordinates the team’s efforts toward approaching positive outcomes) between group positive affect and team creativity [79]. Moreover, Kim & Shin [80] found that cooperative group norms and group positive affect were significant predictors of team creativity, and that this relationship was fully mediated by collective efficacy (i.e., a sense of collective competence shared among team members with respect to responding to specific situational demands and allocating, coordinating, and integrating their resources). Peñalver et al. [81] found full-mediation effect of group social resources (i.e., teamwork, coordination, supportive team climate) between group positive affect and in- and extra-role performance.

As mentioned earlier, a second relevant theoretical framework to explain the relationship between group affect and collective performance refers to the mood-as-input theory [75]. This model states that people use their current mood as an information, and the interpreted meaning and consequences of their mood on their behavior depend on the organizational context in which the mood was formed [82,83]. The model also focuses on the relationship and potential interaction between negative and positive affect to predict creativity. Negative affective tone informs work-unit members that the situation is problematic and leads them to feel the need of carrying actions to remedy the situation [38]. Group negative affect adopts a moderator role (see next section).

Regarding empirical support, 16 out 24 correlations reported in group affect cross-sectional studies proposing a happy–productive relationship are statistically significant and positive (range 0.13 to 0.58); two with member-rated task performance (0.35, and 0.56), four with leader-rated task performance (range 0.13 to 0.58), one with member-rated contextual performance (0.40), two with leader-rated contextual performance (0.14, and 0.20), one with member-rated creativity (0.34), four with leader-rated creativity (range 0.40 to 0.47), and two with objective financial criteria (range 0.19 to 0.43). 

**Group Affect: Third Variables Included in HP Research Models**. Antecedents of group affect found in the literature are related to organizational support climate and leadership. For instance, team climate of support from the organization (i.e., the extent to which team members believe the team is supported by the organization and their managers) was shown to be positively related to positive team mood, which in turn was relating to team performance in a sample of bank branches [39]. Regarding leadership, different studies propose that the leader is a relevant initiator of a particular tone of group affect, which disseminates among members through a contagion process. Leader positive moods led to transformational leadership [45] and positive group affective tone [45,78]. Finally, transformational leadership positively predicts positive group affective tone through team learning goal orientation (i.e., team members’ shared tendencies to develop competence by acquiring new skills and learning from experience) [84].

Moderators. A meta-analysis showed that positive group affect has consistent positive effects on task performance regardless of contextual factors such as group affect source (exogenous or endogenous to the group) and group life span [11]. However, other contextual factors such as group identification, team trust, or the presence of negative affect have proved their influence on the positive group affect–performance relationship. For example, positive group affective tone had a stronger positive influence on willingness to engage in OCB and on perceived team performance when group identification (i.e., the extent to which people define themselves in terms of their group membership) was high [46]. In a second study, positive group affective tone was beneficial for team creativity when team trust was low, and detrimental for team creativity when team trust was high [85]. As seen earlier, negative group affect is an additional boundary condition with the potential to enhance the effect of positive affect on team creativity [38,82]. For example, Tu [83] found that negative affect might be positively related to employee creativity, when contextual factors are supportive (i.e., organizational support is high and organizational control is low) in a sample of 106 new product development (NPD) teams working for high-technology Taiwanese firms. In another study in a Brazilian retail chain, negative affective tone made the relationship between positive affective tone and creativity stronger [38]. The authors contend that negative affective tone may help employees to broaden their modes of creative thinking to identify and solve problems/difficulties. Similarly, Tsai et al. [85] found that positive group affect enhanced creativity when team trust was low and negative group affect was high.

In summary, the theoretical frameworks underpinning the HPWU literature on group affect are the broaden-and-build theory and the mood-as-input theory. Following these theories, positive group affect broadens and activates the teams’ (cognitive, motivational, attitudinal, and behavioral) processes and interactions over a specific period of time leading to improved team performance. Moreover, this literature is starting to take into account the potential interaction of positive and negative group affect and the influence of contextual conditions (e.g., trust, group identification, organizational support, or control) on the HPWU relationship.

**Team Work Engagement Literature: Theoretical Frameworks.** Team work engagement is considered a predictor of collective performance in the nine studies we found in the systematic review. The job demands-resources model of work engagement (JDR-WE model) is the main theoretical framework used to explain why higher levels of engagement lead to increased performance [86]. The JDR-WE model is a model of employee motivation [87]. Its main proposition is that job resources (e.g., social support, autonomy) and personal resources (e.g., self-efficacy, optimism) have a positive impact on engagement, particularly when job demands (e.g., workload, emotional demands) are high. Specifically, challenging job demands (vs. hindering job demands) have the potential to promote employees’ growth and achievement together with their motivation toward the task. In turn, work engagement has a positive impact on job performance. 

The mechanisms operating between team engagement and performance replicate the arguments given at the individual level on how vigor, dedication, and absorption may lead to increased performance. For example, Mäkikangas [88] stated that: “As work engagement is a motivational state characterized by an employee’s will and drive to perform well at work [89], it is reasonable to use it as a predecessor of team job performance” (p.773). A more detailed explanation was offered by Costa [87] who proposed that engaged teams are energetic when working, display active and productive behaviors, are willing to help each other and build on each other’s ideas, and consider their task meaningful and relevant. García-Buades et al. [90] contend that shared team engagement additionally contributes to teams’ performance due to emergent phenomena such as the team members’ alignment towards common goals, increased synergies among members, and better cooperation and interaction processes. The studies identified in the systematic search provide similar arguments about the mechanisms explaining the collective engagement–performance link. However, little research has been conducted on these mechanisms. 

Furthermore, it is worth mentioning some multilevel efforts in the team work engagement–collective performance literature, which contribute to clarify the relationship between team-level constructs and individual level constructs. For instance, individual and team work engagement were associated with high levels of perceived team performance in 102 Finnish teams from the educational sector [88]. Another study found that team work engagement was significantly related to team performance, but it also predicted individual performance through individual work engagement (vigor) [62]. 

Regarding mediators, only one study proposed and found support for service climate and employee performance to mediate an indirect relationship between team engagement and customer loyalty in 114 service units in the hospitality industry [91].

The nine cross-sectional studies proposing a happy–productive relationship based on team work engagement reported 8 out of 11 correlations to be statistically significant and positive (range 0.24 to 0.54); four with member-rated task performance (range 0.30 to 0.54), three with leader-rated task performance (range 0.24 to 0.30), one with member-rated contextual performance (0.38). Although two correlations between team work engagement and customer satisfaction were not significant, results with path analysis found an indirect relationship with customer satisfaction [91] and results with multilevel analyses found a significant effect of team engagement on service performance when climate for innovation was high [90]. 

**Team Work Engagement: Third Variables included in HP Research Models**

Regarding antecedents, a meta-analysis by Christian et al. [7] found that job resources are the most relevant predictor of work engagement. Within the studies identified in the systematic search, team resources arise as relevant antecedents of team work engagement. For example, team resources (supportive team climate, coordination, and teamwork) predicted team work engagement, which in turn predicted performance [58]. Other team resources affecting team work engagement are performance feedback, social support from co-workers, support from supervisor, and information available [87]. In the same study, a direct negative effect of task conflict was found on team work engagement [87]. In another study, transformational leadership increased team work engagement, which in turn enhanced team performance [57].

Another antecedent of team work engagement is team job crafting or collaborative crafting (i.e., the process by which groups of employees determine together how they can alter their work to meet their shared work goals). Team job crafting predicted team work engagement which then predicted leader-rated performance [92], and team-rated performance [62]. In a similar vein, McClelland et al. [93] found support for a model in which collaborative crafting led to three team processes (team efficacy, team control, and team interdependence), which then led to team work engagement and subsequent improved performance in a sample of 242 call centre teams. 

Moderators. Some moderators have been shown to strengthen the influence of team work engagement on performance such as task conflict [87], and climate for innovation [90]. In a study with research teams, Costa et al. [87] found that task conflict may enhance the benefits of engaged teams on objective performance, because engaged teams are more open to discussing new ideas positively and can integrate their members’ contributions better. In another study, multilevel analyses showed significant positive direct relationships between team engagement and service quality indicators in hotel and restaurant units, and a consistent moderating role of climate for innovation—recognition of employees’ ideas and suggestions to improve work methods and the service delivered—so that the relationship between team engagement and service performance became stronger as climate for innovation increased [90]. 

In summary, the main theoretical framework at the base of the HPWU literature on team work engagement is the job–demands–resources model of work engagement. Thus, team resources increase engagement, particularly when challenging demands are high, creating a positive affective-motivational shared state, which leads to improved team performance. This literature emphasizes a varied array of team resources, which increase team work engagement, and in turn enhance team performance. Moreover, it benefits from some examples of multilevel research, which takes into account the effects of team-level well-being and behavioral processes together with individual well-being and performance.

#### 3.2.3. Research Question 3: What Is the Evidence for Causal or Reciprocal Relationships between Collective Well-Being and Collective Performance?

Despite the frequently reported positive significant correlations, the causal relationship between well-being and performance is far from clear. Does well-being increase performance? Or does good performance increase well-being? In the most recent meta-analysis about satisfaction, citizenship behaviors, and performance in work units, Whitman et al. [12] reported the lack of enough longitudinal studies to meta-analytically test causal relationships between collective satisfaction and performance at the unit-level. Therefore, in this section, we first summarize the findings on two meta-analyses on causal HP relationships at the individual and organizational level [94,95]. Then, we describe the findings about causal or reciprocal relationships between collective well-being and collective performance at the work-unit level. 

Two important meta-analyses published at the individual and organizational level provide interesting findings on causal relationships between well-being and performance. At the individual level, Riketta (2008) conducted a meta-analysis of 16 panel studies finding support for job attitudes to increase performance (in-role, extra-role, and objective performance) after controlling for baseline performance, whereas effects of performance on subsequent job attitudes were nonsignificant. Effects of job attitudes on performance were stronger for shorter time lags (less than 6 months compared to longer time lags) suggesting that time lag was a moderator of the cross-lagged relationship. Riketta [94] suggests that attitudes effects may be short lived and recommends exploring shorter spans (e.g., a few days). Furthermore, Riketta [94] found a counterintuitive negative effect of performance on job satisfaction for moderate time lags, which he attributed to people who perform strongly but do not perceive to be adequately rewarded for their performance. Based on these results, he suggests studying the potential moderating role of reward systems and justice perceptions on the job satisfaction–performance relationship (p. 479).

At the organizational level, a meta-analysis by Schneider et al. [95] using data from 35 companies over 8 years showed organizational financial and market performance to be predictors of overall job satisfaction and satisfaction with security more strongly than the reverse. They also reported a more reciprocal relationship of organizational financial and market performance with satisfaction with pay, which they suggest may be mediated by OCB. The authors contend that “the relationship between employee attitudes and organizational performance is complex, and it is too simplistic to assume that satisfaction attitudes lead to organizational financial or market performance—some do and some do not, and some employee attitudes apparently are the result of financial and market performance" (p. 849). They also suggest that non-financial organizational outcomes may show a stronger relationship with satisfaction than financial performance.

Five studies investigated time-lagged or longitudinal HP relationships at the work-unit level, three on collective satisfaction [33,36,37], and two on group positive affect [38,39] (see Table 3 for a summary). In an empirical study, Koys [33] addressed the issue of whether work-unit satisfaction and behaviors (OCB and turnover) influenced business outcomes (profitability and customer satisfaction) in a sample of 28 restaurant units from a chain, and explored the reverse relationship as well. In stressing the relevance of behaviors, he argued that “employee attitudes cannot influence organizational effectiveness on their own; employees must also behave appropriately” (p. 103). Results supported the HP model, in that human resources outcomes (work-unit satisfaction and behaviors) influence work-unit effectiveness, rather than the other way around. More specifically, cross-lagged regression analyses showed that unit-level employee satisfaction, OCB, and turnover measured at year 1, predicted two unit-level profitability measures at year 2 (R^2^ = 0.14, and 0.17), with only OCB having a significant beta weight. The same independent variables predicted customer satisfaction at year 2 (R^2^ = 0.31), with only unit-level employee satisfaction having a significant beta weight. Thus, OCB had an impact on profitability, and employee satisfaction had an impact on customer satisfaction. This research supports the idea that unit-level employee satisfaction leads to OCB, which in turn leads to profitability; and additionally, employee satisfaction leads to customer satisfaction.

Messersmith et al. [36] studied the link between high-performance work systems (HPWS) and time-lagged performance in a sample of 119 public service departments in Wales. Their results support a research model in which department-level HPWS enhanced what they called the black box (employee attitudes–job satisfaction, organizational commitment, psychological empowerment, and behaviors (OCB)), which was further related to objective departmental performance. The authors highlight the important role that aggregate discretionary behaviors (OCB) may play in the success of departmental units. HPWS initiate a chain in which employees are more likely to engage in the prosocial behaviors that help organizational units to meet goals and objectives. In combination, these helping behaviors allow organizational units to be more efficient and flexible, as “employees are more likely to step beyond the bounds of their narrowly defined job descriptions to assist each other as well as to help maximize their overall departmental functions. In addition, this reciprocity is likely to have continual positive effects in the department as OCBs become enmeshed as a part of the established norms and values in the culture of the unit” (p. 1114).

A third study explored the longitudinal relationships between work-unit climate and labor productivity in a sample of 171 bank branches in the Netherlands [37]. Based on previous findings at the individual level, the main hypothesis was that two climate types (goals and service orientation) would positively influence (objective) productivity through increased work-unit satisfaction. The rationale is that shared positive attitudes would be a prerequisite for engaging in collaborative effort and accepting organizational goals. They also explored the reversed causation model in which productivity would increase the branch resources to invest in their employees, resulting in higher positive climate scores and work attitudes. Contrary to their expectations, they found no evidence for work satisfaction as an intermediary mechanism at the business level, and additionally they found no effects between work satisfaction and productivity. Thus, they concluded that the happy–productive thesis valid at the individual level, does not obtain empirical support at the branch-level. They attributed these results to the sample (business sector instead of multiple or educational), to the lower associations usually found for longitudinal rather than cross-sectional associations (average time lag was 2 years), and to the use of aggregated work satisfaction. They suggest that “at branch level, more active concepts like OCB or work engagement may be more strongly related to unit performance compared to traditional attitudinal concepts like work satisfaction” (p. 306). 

Two time-lagged studies explored the relationship between group affect and collective performance. In a study with Spanish bank branches, a team climate of support from the organization was positively related to positive team mood, which in turn was positively related to team members’ ratings of team performance but not to leader-rated team performance [39]. Another study explored how store positive affective tone predicted store performance (i.e., sales achievement) in a sample of 94 Brazilian retail stores [38]. Although the correlation and direct path between positive affect and store performance were non-significant, results showed that positive affective tone predicted the stores’ performance through the mediating role of creativity, even after controlling the effects of preceding stores’ performance. Thus, “happier” stores were more creative and more effective (i.e., increased sales).

Two of these studies additionally explored a reverse causality direction [33,37] from performance (Time 1) to happiness (Time 2). Koys [33] found a stronger correlation between collective work-unit satisfaction (Time 1) and customer satisfaction (Time 2) (r = 0.61), than the reverse (r = 0.36). He also found stronger correlations between collective work-unit satisfaction and two profit measures (r = 0.35; r = 0.27), than the reverse (ns). On the other hand, he also reported a stronger correlation for manager-rated OCB (Time 1) and collective satisfaction (Time 2) (r = 0.32), than the reverse (ns). Van de Voorde [37] reported cross-lagged correlations between objective indicators of performance (financial branch yearly productivity index) and collective satisfaction in two directions, both were nonsignificant.

In summary, four out of five studies exploring causal relationships between collective well-being and collective performance at the unit level provide empirical support to a happy–productive direction. Three of them obtain support for a causal chain between unit-level well-being (work-unit satisfaction or group positive affect), unit-level behaviors (OCB or team creativity), and objective unit-level performance (profitability, departmental performance, and sales respectively) [33,36,38]. These three studies propose a causal chain in at least three steps “well-being–team performance–objective performance”. Interestingly, the fourth study proposed a direct link between well-being and objective performance, without considering a “team behavior” as a mediator, and found no empirical support for the collective satisfaction–performance link. Overall, the very limited existing evidence suggests that collective well-being would enhance relevant discretionary team behaviors (OCB and creativity), which in turn increase objective productivity.

## 4. Discussion

The aim of this systematic review was to offer an integrated overview on the literature on happy-productive work-units (HPWU). More specifically, we reviewed quantitative peer-reviewed studies published between 2001 and 2018 on the relationship between collective happiness and collective performance for teams and work-units. With our systematic review, we found 30 empirical studies on HPWU, which adopted three main collective well-being constructs and four categories of collective performance. Research has focused on hedonic well-being (satisfaction, 7 studies; group Affect, 14 studies), and more recently on eudaimonic well-being (teamwork engagement, 9 studies). We could not identify research on other eudaimonic constructs applied to teams (e.g., collective flourishing, purpose or meaning of work), which could, we believe, further explain extra-ordinary team efforts and success (e.g., such as the world-watched rescue of 12 soccer players from a flooded cave in Thailand in July 2018). On the other hand, collective performance relies mainly on team performance (task performance, OCB, creativity; supervisor-rated and member-rated), and less so on customer satisfaction or financial/objective ratings. The literature on HPWU is scarce, diverse, and disintegrated. There is diversity both in the operationalization of collective well-being and performance and in the theoretical frameworks used.

Our main contribution is the analysis of the strengths, commonalities, and differences across these three well-being literatures to get a comprehensive picture of what is known on happy–productive teams. To do so, we reviewed and summarized how collective well-being and performance is measured, the theories that attempt to explain how happy teams become productive teams or productive teams become happy, and whether both collective phenomena reciprocate. In this section, we summarize and discuss the results following the research questions set out for the review, reflect on the limitations of our study, and suggest avenues for future research. 

### 4.1. Discussion

#### 4.1.1. Research Question 1—Which Are the Main Features of the Conceptualization and Operationalization of Collective Well-Being?

The HPWU literature builds on diverse conceptualizations and operationalizations of well-being and performance. A question that remains open is about the relationship among the three constructs of collective well-being and their relative contribution to collective performance. Far from addressing this issue, the reviewed literature about collective well-being mostly ignores it. An exception is the work by Mason & Griffin [35], who differentiated and explored the effects of group task satisfaction and group affective tone on group outcomes. They found “group task satisfaction” to explain unique variance in civic helping and sportsmanship behavior, but not in leader-rated team performance, once the effects of aggregated “individual job satisfaction” and group affective tone had been taken into account. Second, the literature on positive group affect considers team satisfaction as a mediator between group affect and performance, therefore placing group affect as an antecedent of satisfaction. Positive group affect has been considered as an antecedent of both satisfaction [45] and engagement [6,7]. Third, literature on engagement proposes satisfaction as either an antecedent or a consequent of engagement [6]. Although engagement and satisfaction have shown considerable overlap, enough discriminant validity between them justifies their differentiation [7]. Overall, only good quality, longitudinal research studying simultaneously group affect, group satisfaction, and team engagement will be able to elucidate the relationship among different types of well-being, whether they overlap or complement each other, and their relative contribution to performance.

Regarding collective performance, the overreliance on subjective effectiveness measures is an additional weakness of existing research. Although team-member and leader-rated team performance are valid and useful measures of performance, studying more distal (e.g., customer satisfaction) or objective team performance criteria would add extra value to future research. Task and contextual team performance are the main focus of collective performance by the three well-being literatures. A particular strength of the collective satisfaction literature is evaluating performance not only through team performance (task performance and OCB) but also through distal team effectiveness outcomes (i.e., customer satisfaction and/or financial performance). Besides, only group affect researchers study team performance by means of team creativity. Using a combination of multiple valid operationalizations of collective well-being and collective performance would contribute to a more comprehensive picture of the HPWU relationship and allow for sound comparisons among HP constructs. 

Regarding operationalizations of collective well-being, despite some valuable contributions in defining and measuring work-unit satisfaction [12,35], they had limited influence both on subsequent research and extending the use of valid group-satisfaction measures. However, significant results obtained across diverse operationalizations support the consistency of the relationship between collective satisfaction and performance. Alternatively, operationalizations of group affect and team engagement are more homogeneous and uniform than measurement of satisfaction, thus allowing for comparability among studies and results. 

In general, recommendations for appropriate measurement of team-level well-being includes use of valid instruments, use of team referents (vs. individual referents), and use of multiple informants (e.g., leaders, peers). Furthermore, collective well-being ratings generally rely on the aggregated unit’s average score. This method emphasizes sharedness of well-being, a core quality of collective well-being, however there are calls to investigate the effects of the strength of well-being among team-members and over time. Additionally, an issue of substantial interest is to examine the amount of variability and dispersion of work-unit well-being within a team and its influence on collective performance [12,96].

Overall, the variety in definitions and operationalizations of collective constructs make it difficult to reach consistent conclusions about HP relationships but it provides a more comprehensive and holistic view on these relationships.

#### 4.1.2. Research Question 2—Which Theoretical Frameworks Are Used to Explain the Collective Happy–Productive Work Unit Thesis? 

The theoretical frameworks on the HPWU relationship and mechanisms linking collective well-being and collective performance are specific for each body of literature. Regardless of the consideration of well-being as hedonic or eudaimonic, it is implicitly or explicitly assumed that well-being shared among team-members is a relevant antecedent of collective performance. In this section, we reflect on the strengths of different theoretical efforts to explain how collective well-being influences collective performance and the mechanisms through which shared well-being translates into better team performance. 

The literature on team satisfaction builds on the general research on attitudes and behavior. The predominant theoretical framework expands the happy–productive thesis to the team level, complemented with additional theoretical models like social exchange theory, linkage research, or the service–profit chain. The rationale behind the HPWU thesis is that when collective satisfaction emerges as a shared phenomenon, it activates collaboration and OCBs among team members (i.e., a measure of team performance/processes), which subsequently leads to improved (objective) performance. Thus, team satisfaction facilitates collaborative effort towards, and acceptance of, organizational objectives. The literature in satisfaction proposes and finds empirical support for “OCB” as a key mediator between collective well-being and objective/financial performance, following a causal chain “collective satisfaction–team performance (OCB)–financial performance” [12,33,97]. 

The literature on group affect showed a strong focus on uncovering the mediating mechanisms between well-being and performance. Building on the broaden-and-build theory [74], the rationale is that shared positive group affect activates relevant cognitive (e.g., goal setting and reviewing), motivational (e.g., goal commitment), attitudinal (e.g., team satisfaction), and behavioral (e.g., providing help and support) group processes, which subsequently improve performance. Existing cross-sectional research finds empirical support for these mediating mechanisms. Based on the mood-as-input model [75], another relevant contribution of the group affect literature is the study of the combined effect or interaction between positive group affect and negative group affect on team’s performance (e.g., mainly team creativity). Negative group affect has been shown to enhance the positive effects of positive group affect on team performance in particular situations in which the team needs to solve problems or face difficulty [38].

Research on team work engagement benefits from a sound theoretical framework (i.e., the job demands–resources model of work engagement, JDR), a precise concept, and well-established instruments. Research linking team work engagement and collective performance argues that a balance between team resources and challenging demands promotes energized team members, who consider their task meaningful and show active, productive, and helpful behaviors. Research interest in the engagement literature emphasizes the identification of team resources as antecedents of well-being (transformational leadership, resiliency, collective efficacy).

The reviewed studies offer empirical evidence of the relevance of multiple antecedents of well-being, and moderators of the HP collective relationship. Regarding antecedents, the role of leadership seems especially relevant, in particular transformational and charismatic leadership behaviors have shown a significant effect on a work unit’s collective well-being and behavior [12,45,57]. Other antecedents include organizational practices (HPWS or climate), work attributes (team task characteristics), and team resources of multiple kinds. Several moderators stand out as particularly important in the literature reviewed on HPWU such as sharedness (or strength of well-being), sector, team size, team tenure, and third variables such as group identification, team trust, negative affect, and shared job crafting. Further research is needed to clarify and compare the roles of antecedents, mediators and moderators of the HPWU relationship in order to guide interventions and investments [14]. 

#### 4.1.3. Research Question 3—What Is the Evidence for Causal or Reciprocal Relationships between Collective Well-Being and Collective Performance?

The predominant view in previous research and across the 30 HPWU studies identified in our review is that collective well-being is an antecedent of collective performance. The happy–productive relationship obtains generalized empirical support as a positive and significant relationship both in previous meta-analyses and in our findings. Previous meta-analyses have provided moderate corrected correlations between work-unit satisfaction and performance (r = 0.34) [12], group affect and group task performance (r = 0.33) [11] and business-unit engagement and performance (r = 0.42) [55]. In our systematic review, we found 76.2% of the correlations reported were positive and significant across different constructs of collective well-being and performance. Existing evidence so far shows a moderate relationship, does not allow concluding causal direction, and is affected by mediating and moderating third variables. However, these results heavily rely on correlational and cross-sectional studies, and thus the causal direction between different types of well-being and different types of performance remains an open question. 

Two meta-analyses on longitudinal relationships between well-being and performance are available at the individual [94] and organizational [95] levels of analyses, with none available at the work-unit level due to the dearth of longitudinal studies [12]. Overall, their results show weaker or non-significant correlations between collective well-being and time-lagged measurements of performance compared to cross-sectional measurements. Shorter time-lags (less than 6 months) yielded stronger relationships between job satisfaction and performance at an individual level meta-analysis [94]. Careful selection of time-lags between measurements of collective satisfaction and performance may shed light as to whether the effects of collective well-being vary short-term vs. long-term. Another time-related question is whether some collective well-being constructs have longer impact on performance than others. For instance, it has been suggested that engagement is associated with the development of resources and the pursuit of long-term goals [98].

Furthermore, stronger correlations in the direction of well-being to performance than the reverse were found at the individual level [94], and mixed evidence on the direction HP or PH at the organizational level depending on the specific measure of satisfaction used [95]. Only five studies analyzed time-lagged HP relationships, and only two reported correlations in the reverse direction (PH). The limited but very interesting findings of these five studies showed that collective well-being would lead to relevant discretionary team behaviors (OCB and creativity), which in turn increased objective productivity. Overall, the question remains open as to whether well-being leads to performance or the reverse is true until more research with time-lagged or longitudinal designs is conducted. Future research should undertake panel studies at the work-unit level with improved methodological quality to be able to shed light on issues of causality or reciprocity between collective well-being and collective performance.

### 4.2. Implications for Practice

The findings from this systematic review suggest several implications for practice on how to increase collective well-being, and how to enhance transformation of collective well-being into collective performance. Based on the empirical evidence reviewed, strategies to improve collective well-being should focus on relevant antecedents such as implementing HPWS, encouraging transformational and charismatic leadership, allowing for collaborative job crafting, improving team work design, promoting organizational support climate, and providing team resources. Building on the mediating mechanisms identified, it is also recommended to train team leaders and members on team work processes (e.g., team reflexivity, team helping behavior, OCB) and on how to maintain positive interactions (e.g., building on others’ ideas, morale-building communication). Finally, attending to (instead of ignoring) important moderators such as negative affect or task conflict help teams to focus efforts on relevant team problems and avoid defective team decision-making.

### 4.3. Recommendations for Future Research

#### 4.3.1. Antagonistic Patterns of HPWU

The relationship between well-being and performance is more complex than the HPWT proposes and needs to be re-defined [9]. Beyond happy-productive workers or teams (synergistic pattern), alternative and antagonistic patterns of happy–unproductive or unhappy–productive teams should be considered. For instance, Peiró et al. [9] found that over 50% of the respondents were classified in antagonistic patterns in a sample of Spanish workers. Furthermore, they found that the same employee belonged to different patterns of well-being and performance in different operationalizations of well-being (hedonic vs. eudaimonic) and performance (self-rated vs. supervisor rated), e.g., the same employee could be hedonically happy, eudaimonically unhappy, and receive high or low performance ratings depending on the source of evaluation. At the team level, HPWU research emphasizes the positive influence of well-being on valuable performance outcomes such as task performance [11,12], team creativity [38,79,83], and customer satisfaction [33,70]. However, evidence exists also on the negative consequences of being a happy team (defective decision-making, group-think, social loafing). Thus, applying an expanded HPWT model with synergistic and antagonistic patterns to the study of teams and work-units would be a fruitful avenue for future research. Researchers might, for example, investigate the conditions under which a team transitions from one happy–productive pattern to another pattern over time; explore team composition to understand how individual members’ HP patterns contribute to a team’s general HP pattern; study which top-down (e.g., leadership) or bottom-up factors (e.g., team processes) enhance team’s happiness and productivity. To address these questions, we believe both qualitative and quantitative research is strongly needed to uncover the dynamic relationship between collective well-being and performance. 

Relationship between hedonic and eudaimonic well-being. A relevant question for future research is the relationship between hedonic well-being and eudaimonic well-being. In other words, can team members be hedonically satisfied and not feel engaged? Can team members feel engaged while experimenting negative affect or dissatisfaction? The likely answer is “yes” they can, but additional questions arise. How long can a team stay in a situation in which eudaimonic well-being is present but hedonic well-being is absent? How do they move or which dispositional or situational factors help the team stay in one state or change from one well-being form to another? This issue is related to team dynamics and transitions over time.

#### 4.3.2. Gain Spirals

There is too limited longitudinal evidence to draw conclusions about the causal ordering of team happiness and productivity. Most likely collective well-being and collective performance maintain a reciprocal and dynamic relationship as teams and team-members move through affective states and performance episodes. Previous research has found empirical support for gain spirals between resources and work engagement [99], and between work engagement and performance indicators like financial returns [100]. A gain spiral is a cycle of positive, mutual reinforcement relationship among constructs and an increase in their levels over time. We believe this theme could be expanded to the study of the HPWU relationship to investigate whether reciprocal relationships between collective well-being and performance may lead to positive cycles and upward spirals, or to negative cycles and downward spirals. Moreover, researchers might identify key third variables with an impact on these spirals such as collective efficacy or conflict management.

#### 4.3.3. Situational and Personal Features

There may be other factors, beyond the work environment, that could affect work performance and well-being. For example, employee impaired health can be critical for organizations, because workers with worse health often lose more work hours, ask for more sick leave, and are resultantly less productive than healthy workers [101]. Recent studies on the HPWT suggest to identify situational and personal features that are associated with adscription to different happy-productive clusters [9,14]. Current research has considered situational features related to job characteristics such as task demands [69] or supervisor behavior [45,102]. Future research should consider other situational features (e.g., time pressure, availability of feedback) as well as personal and social factors related to team members. For instance, factors such as individual health status and chronic diseases [103], age [104], gender [9], educational qualification [105], occupational category [9], and cultural background [106] have shown to have an influence on well-being and/or performance. Further research on the effect of these third variables on patterns of well-being and performance would enrich the knowledge about happy-productive teams.

#### 4.3.4. Multi-Level Methodology

The use of a multilevel methodology would be highly recommended to explore cross-level relationships among organizational, unit-level, and individual constructs. For instance, Tims et al. [62] found support for team work engagement to influence individual work engagement and individual performance. Mäkikangas et al. [88] found support for shared job crafting as a cross-level moderator so that in teams with high levels of shared job crafting engaged individuals perceived better team performance. In a longitudinal study on the role of workforce engagement to predict organizational financial and customer satisfaction, Schneider et al. [54] found that organizational practices (e.g., senior leadership communication of company’s goals) were surprisingly the strongest correlate of workforce engagement compared to supervisory support and work attributes. Further research could explore how organizational level variables influence and promote work-unit well-being and performance (e.g., HPWS).

#### 4.3.5. Methodological Issues

Some recommendations on methodological issues can be drawn from previous research [94,95]. Recommendations would include using consistent, valid, and multiple measures of well-being and performance; careful selection of administration procedures and time lags between measurements; control for sectors, countries, and industry effects; and consider aggregation issues beyond the unit’s average. Furthermore, another point of interest would be to widen the size and diversity of samples. Whitman [12] suggests to increase the number of teams over 400 units, but it is also important to research teams and workgroups in different conditions such as virtual or disperse teams, teams with short vs. long term belonging members, teams with frequent membership changes or with multiple supervisors. Investigating how well-being develops among these teams (e.g., virtual teams, or with frequent membership changes) and how this relates to team performance would be an interesting avenue for future research. 

### 4.4. Limitations

There are important limitations to consider within the literature identified through the systematic review. First, there is a dearth of research on eudaimonic constructs beyond team engagement, and theoretically relevant perspectives such as Kahn’s conceptualization are not developed at the team/work-unit level. Second, collective performance relies on single measurements, one-faceted, and mainly on subjective evaluations of team performance rather than objective measurements. Third, most studies rely on cross-sectional designs to test proposed causal models. 

At review level, there are some limiting factors worth mentioning. The focus of our review was on happy–productive teams and work-units. Therefore, we adopted a positive perspective and excluded concepts that indicate ill-being or lack of well-being such as dissatisfaction, burnout or negative affect just to name a few. Obviously, teams and work-units go through bad times and the management of dissatisfaction, conflict, and frustration may be more important than the management of well-being. Previous research has studied how dissatisfaction or negative affect influences group performance, and found promising results on how negative well-being can lead to improved performance under certain conditions [11]. However, studying unhappy teams was out of the scope of our review. We also excluded team outcomes not directly related to productivity such as absenteeism or turnover. As Schneider et al. [95] pointed out, it may be that satisfaction shows stronger relations with other outcomes rather than financial productivity. We have focused on refereed studies to ensure a certain level of quality in the empirical evidence reviewed. This may have left out interesting unpublished research and to a certain extent bias our review with shortcomings associated with published research (i.e., over-representation of significant results and lack of replication studies). In this line, publication of studies with non-significant results and replication studies would make it possible to accumulate enough and better evidence to progress knowledge and understanding on HPWU. Besides, the scarce number of studies that focus on the relationship between well-being and performance at the collective level of analysis coupled with the heterogeneity among these studies impede carrying out a meta-analysis that would have helped to further integrate the results. Once we dispose of enough accumulated studies on this relationship, we suggest future research to perform a meta-analytic study on this topic in order to add valuable information, in particular about causality.

## 5. Conclusion

Despite all its limitations, literature on collective well-being has contributed to understanding the relevant role of affective processes happening in teams and work groups, which have largely been ignored compared to cognitive processes. Theoretical frameworks underpinning the collective well-being–performance relationship are mainly the happy-productive worker thesis and general attitude-behavior link for collective satisfaction; the broaden-and-build theory and input-as-mood theory for group affect; and the job-demands-resources model and broaden-and-build theory for team engagement. The positive effects of collective well-being (or affective climates) on team performance, team creativity, customer satisfaction, and financial performance have been empirically tested and found throughout the studies included in this systematic review. Across a diversity of samples, organizational sectors, countries, conceptualizations, and operationalizations of well-being and performance, collective well-being is positively correlated to collective performance at the meso-level of analysis (team, group, work-unit). In particular, the satisfaction and group affect literatures have shed light on mediating team processes that explain the link between collective well-being and performance. Additionally, literature found on the three collective well-being constructs offers very interesting inputs on antecedents (e.g., transformational leadership, resources, task characteristics), and moderators of the HPWT (e.g., strength of well-being, group identity, negative group affect, organizational support) at the unit-level.

## Figures and Tables

**Table 1:** Hedonic and eudaimonic perspectives on individual and collective well-being at work

Happiness	Individual Happiness at Work	Collective Happiness at Work
Hedonic	Affect Emotions Mood Job satisfaction	Group affect Group mood Collective satisfaction Group task satisfaction
Eudaimonic	Work engagement Flow Meaning at work Flourishing Personal growth	Unit-level engagement

**Table 2:** Theoretical frameworks linking collective well-being and collective performance.

Collective Well-Being	Defined as	Theoretical Frameworks	Mechanisms Linking Well-Being and Work Performance	Most Popular Measures
Team Satisfaction	A shared attitude (or shared positive emotional state) towards the team task and environment	Happy productive thesis Human relations school Social exchange theory Linkage research Service-profit chain	Attitude–behavior link: Facilitates collaborative effort, acceptance of goals, interactions and dependencies	Aggregated Job Satisfaction Group task satisfaction
Group Affect	Positive affect while on the job or during team meetings (transient mood)	Broaden-and-build theory Mood-as-input model	Improves specific team processes: cognitive, motivational, attitudinal, behavioral	Positive Affect (PANAS) Emotion scales
Team Work Engagement	Positive, fulfilling, work-related shared state of vigor, dedication, and absorption	Job-demands-resources model of work engagement Broaden-and-build theory	Motivational process triggered by job resources and demands	UWES: Utrecht Work Engagement Scale (for teams) Team Work Engagement Scale

**Table 3:** Summary of time-lagged correlations between happy–productive teams and productive–happy teams.

**Happy–Productive**	**T1**	**T2**	**r**	**Time Lag**
González-Romá et al. (2012)	Team positive mood	Team performance	0.39 **	1 year
	Team positive mood	Team effectiveness	0.21 ns	
Koys (2001)	Satisfaction	Manager rated OCB	0.19 ns	1 year
	Satisfaction	Profit sales	0.35 *	
	Satisfaction	Profit year 2	0.27t	
	Satisfaction	Customer Satisfaction	0.61 *	
Messersmith et al. (2011)	Job Satisfaction	Department performance	0.36 *	1 year
	Job Satisfaction	Self-rated OCB	0.36 *	
Rego et al. (2013)	Positive affective tone	Performance subsequent semester	0.07 ns	6 months
Van de Voorde et al. (2014)	Satisfaction	Productivity	0.06 ns	Average 2 years
**Productive–Happy**	**T1**	**T2**	**r**	**Time lag**
Koys (2001)	Manager rated OCB	Satisfaction	0.32 *	1 year
	Profit Sales	Satisfaction	0.15 ns	
	Profit Year	Satisfaction	0.05 ns	
	Customer Satisfaction	Satisfaction	0.36 *	
Van de Voorde et al. (2014)	Productivity	Satisfaction	0.02 ns	Average 2 years

Note. * *p* < 0.05. ns not significant.

## Supplementary Materials

The following are available online at https://www.mdpi.com/1660-4601/17/1/69/s1.

## Appendix A

**Table A1:** Description of the studies on Collective Satisfaction – Collective Performance.

Source	Study Goal	Theories	HP ^a^	JS Definition ^b^	JS Measure	Global/Facets ^c^	JS Informant JS Referent	P Measure ^d^	Design ^e^	R ^f^	Sample	Team Size
1. Chi, Chung, & Tsai (2011) [45]	Examine two mediating mechanisms that explain the leader positive moods–team performance linkage: transformational leadership, and positive group affective tone	Input-process-output model of teams (Hackman, 1987); Emotional contagion (Hatfield et al., 1994); Broaden-and-build theory (Fredrickson, 1998)	HP	Team satisfaction is an attitudinal construct that reflects a team’s shared attitude toward team tasks and their associated environments (Mason & Griffin, 2005)	Team satisfaction (3- items; Barsade et al., 2000)	G	Team members; Individual	Performance scale (4-items; Edmonson, 1999). Supervisors. Subjective	C	0.27 *	85 sales teams. Taiwan	7.34
								Performance scale (4-items; Edmonson, 1999). Employees. Subjective		0.46 **		
								Objective performance (first-year commission, first-year premium, total commissions, team goal achievement). Supervisors. Objective		0.29 **		
2. Koys (2001) [33]	Examine whether positive employee attitutes and behaviours influence business outcomes or whether positive business outcomes influence positive employee attitudes and behaviors	Attitude- cooperation collaboration- unit productivity (Ryan et al., 1996); climate for service (Schneider et al., 2005); service profit chain (Heskett et al, 1997); social exchange theory (Blau, 1964; refering to OCB)	HP	n/a	Employee satisfaction (4-items; Foodservice Research Forum, 1997)	G	Employees; Individual	OCB (5-items; Organ, 1988). Leader. Subjective	CL (1 year)	JS(T1)-OCB(T1) = 0.47 **; JS(T1)-OCB(T2) = 0.19ns; JS(T2)-OCB(T2) = 0.61 **	28 restaurant units. n/a	28 (T1); 25 (T2)
								Customer satisfaction (4-items; n/a). Customers. Subjective		JS(T1)-CS(T1) = 0.49 **; JS(T1)-CS(T2) = 0.61 **; JS(T2)-CS(T2) = 0.09ns		
								Profit year (company records). Leader. Objective		JS(T1)-PY(T1) = 0.10ns; JS(T1)-PY(T2) = 0.27; JS(T2)-PY(T2) = 0.22ns		
								Profit sales (company records). Leader. Objective		JS(T1)-PS(T1) = 0.37 *; JS(T1)-PS(T2) = 0.35 *; JS(T2)-PS(T2) = 0.43 **		
			PH					OCB (5-items; Organ, 1988). Leader. Subjective		OCB(T1)-JS(T2) = 0.32 *		
								Customer satisfaction (4-items; n/a). Customers. Subjective		CS(T1)-JS(T2) = 0.36 *		
								Profit year (company records). Leader. Objective		PY(T1)-JS(T2) = -0.05ns		
								Profit sales (company records). Leader. Objective		PS(T1)-JS(T2) = 0.15ns		
3. Li, Li, & Wang (2009) [69]	Explore the relationships among traditional task characteristics, team performance and team member satisfaction	Input-process-output model of teams (Hackman, 1987). Social Exchange Theory (Blau, 1964; refering to OCB)	HP	n/a	Team member overall satisfaction (2-items; Cohen, 1996)	G	Team members; Individual	Overall team performance (5-items; Rosenstein, 1994). Team members. Subjective	C	0.52 **	92 teams. n/a	3.82
								Overall team performance (5-items; Rosenstein, 1994). Managers. Subjective		0.32 **		
4. Mason & Griffin (2005) [35]	Test the validity and utility of group task satisfaction and investigate whether group task satisfaction would explain incremental variance in organizational citizenship behaviors, group performance, and absenteeism norms, after the variance explained by aggregated individual job satisfaction and group affective tone was taken into account	Emotional contagion (Hatfield et al., 1994). Atraction-selection-attrition effects. Social information	HP	The group’s shared attittude towards its task and the associated work environment (Mason & Griffin, 2002)	Group task satisfaction (10-items; Mason & Griffin, 2005)	F	Group members; Team	OCB (civic helping, sportsmanship; Podsakoff et al., 1997). Group members. Subjective	C	0.32 * (civic helping) 0.43 ** (sportmanship)	55 work groups variety of industries. Australia	9.32
								OCB (civic helping, sportsmanship; Podsakoff et al., 1997). Supervisors. Subjective		0.20ns (civic helping) 0.21ns (sportmanship)		
								Group performance (quality, customer service, productivity; n/a). Supervisors. Subjective		0.19ns		
					Individual job satisfaction (MSQ; 20-items; Weiss et al., 1967)		Group members; Individual	OCB (civic helping, sportsmanship; Podsakoff et al., 1997). Group members. Subjective		0.35 ** (civic helping) 0.32 * (sportmanship)		
								OCB (civic helping, sportsmanship; Podsakoff et al., 1997). Supervisors. Subjective		0.22ns (civic helping) 0.21ns (sportmanship)		
								Group performance (quality, customer service, productivity; n/a). Supervisors. Subjective		0.31 *		
5. Messersmith, Patel, Lepak, & Gould-Williams (2011) [36]	Explore potential individual attitudinal and behavioral mediators aggregated at the unit level that operate in the black box between HR systems and departmental performance	Social exchange theory (Blau, 1964)	HP	n/a	Satisfaction (3-items; Bowling & Hammond, 2008; Spector et al., 1999; Vancouver & Schmitt, 1991)	G	Department members; Individual	Departamental performance (% success). Managers. Objective	CL (1 year)	0.36 *	92 departments local government authorities. Wales (United Kingdom)	148
								OCB(8-items; Smith et al., 1983). Employees. Subjective		0.21 *		
6. Namasivayam, Guchait, & Lei (2014) [70]	Examine the role that psychological empowement and employee satisfaction play in the relationship between leader empowering behaviors and customer satisfaction and employees’ organizational commitment	Linkage research (Schneider et al, 2005). Service-profit chain (Heskett et al, 1997)	HP	n/a	Satisfaction (2-items; Hirschfield, 2000)	G	Frontline employees; Individual	Customer satisfaction (6-items; n/a). Customers. Subjective	C	0.57 *	40 restaurant units. Northeastern US	n/a
7. Van de Voorde, Van Veldhoven, & Paauwe (2014) [37]	Test the mediating role of work satisfaction in the relationship between work unit climate and labour productivity	Satisfaction-behavior-productivity (Kopelman, Ostroff); climate-attitudes-performance (Schneider et al., 2005); service profit chain (Heskett et al, 1997)	HP	Group task satisfaction as the group’s shared attitude towards its task and the associated work environment (Mason & Griffin, 2002)	Job satisfaction (10-items; Bakker et al., 2010)	G	Employees; Individual	Labour productivity (profits-to-costs ratio). Finance and control department. Objective	CL (1-4 years)	JS(T1)-LP(T1) = 0.08ns	171 financial services branches. Netherlands	84.7 (T1); 86.9 (T2)
										JS(T2)-LP(T2) = 0.17 *		
										JS(T1)-LP(T2) = 0.06ns		
										JS(T2)-LP(T1) = 0.02ns		

^a^ HP = Happy-Productive; PH = Productive-Happy; ^b^ JS = Job Satisfaction; ^c^ G = Global; F = Facets; ^d^ P = Performance; ^e^ C = Cross-sectional design; CL = Cross-lagged design; ^f^ R = correlation coefficient; ns = not significant; * = *p* < 0.05; ** = *p* < 0.01; *** = *p* < 0.001.

**Table A2:** Description of the studies on Group Affect – Collective Performance.

Source	Study Goal	Theories	HP ^a^	GA Definition	GA Measure ^b^	GA Informant GA Referent	P Measure ^c^	Design ^d^	R ^e^	Sample	Team Size
1. Boerner & Freiherr von Streit (2007) [50]	Investigate the degree to which a conductor’s transformational leadership and orchestral musicians’ positive group mood have a beneficial effect on orchestral performance	n/a	HP	Work group mood (Bartel & Saavedra, 2000) or group emotion (Barsade & Gibson, 1998) is a specific disposition developed through processes of cognitive and emotional self-regulation among group members	Group mood (8-items; Boerner & Freiherr von Streit, 2007)	Employees; Group	Artistic quality (Auvinen, 2001): (1) the reaction of third parties to the orchestra’s achievement (2) Quality compared to other orchestras of the same quality). Two members. Subjective	C	0.56 **	22 Symphony orchestras. Germany	≤ 12
2. Chi & Huang (2014) [84]	Explore the mechanisms that explain the relationship between transformational leadership and team performance	Three-stage model of transformational leadership (Conger & Kanungo, 1998)	HP	Group affective tone reflects team members’ affective reactions toward current team conditions (George & King, 2007)	(1) PANAS (n/a; Watson et al., 1988) (2) team members evaluation the extent to which each of a list of adjectives described their mood states at team meetings during the past week (e.g., Tsai et al., 2012)	Team members; Individual	Team performance scale (5-items scale; Edmondson, 1999). Supervisors. Subjective	C	0.58 **	62 teams. High-technology firms. Taiwan	4.5
3. Chi, Chung & Tsai (2011) [45]	Examine how transformational leadership, and positive group affective tone mediate the relationship between leader positive moods and team performance	Input-process-output model of teams (Hackman, 1987); Emotional contagion (Hatfield et al., 1994); Broaden-and-build theory (Fredrickson, 1998)	HP	Positive group affective tone is defined as the homogeneous positive affective states within the group (George, 1990)	PANAS (10-items; Watson et al., 1988). Past two weeks. Team meeting	Employees; Individual	Performance scale (4-items; Edmonson, 1999). Supervisor and employees. Subjective	C	0.30 **(supervisor); 0.35 **(employees)	85 teams. Insurance firms. Taiwan	7,34
							First-year commission, first-year premium, and total commissions earned by the team. Supervisor. Objective		0.36 **		
4. González-Romá & Gamero (2012) [39]	Test whether the relationship between a team climate of support from the organization and team performance is mediated by positive team mood	Motivational control theory (Hyland, 1988; Klein, 1989)	HP	Positive team affect refers to the positive moods shared by team members (Gamero, González-Romá, & Peiró, 2008)	Affective well-being scale (6-items; Segura & González-Romá, 2003). Over the last weeks	Employees; Individual	Group performance scale (2-items; Jehn et al., 1999). Employees. Subjective	CL (1 year)	Team positive mood(T2)-Team performance(T3) = 0.39 **	59 branches.Saving banks. Spain	4.42 (T2 and T3)
							Team effectiveness (1-item; n/a). Supervisor. Subjective		Team positive mood(T2)-Team effectiveness-(T3) = 0.21ns		
5. Kim & Shin (2015) [80]	Examine cooperative group norms and group positive affect as antecedents of team creativity and explore collective efficacy as an intermediary mechanism between these relationships	Social cognitive theory (Bandura, 1986); Group creativity process model (Dzindolet, 2008); Broaden-and-build theory (Fredrickson, 1998)	HP	Group affective tone is defined as consistent or homogeneous affective reactions within a group (George, 1990)	PANAS (4-items; Watson et al., 1988). At work	Team member; Individual	Creative performance scale and creativity scale (6-items; Oldham & Cummings, 1996; Zhou and George, 2001). Supervisor. Subjective	C	0.40 ***	97 teams. Different organizations (eg. service, backing and financial service, manufacturing, and other). Korea	6,1
6. Kim, Choi & Lee (2016) [82]	Examine the moderating role of group affective climate and group reflexivity in the relationship between trait affect and creativity	Mood-as-input model (Martin et al., 1993)	HP	n/a	Positive and negative affective climate (Haslam, 1995). In general. At work	Employees; Group	Employee creativity scale (6-items; Zhou & George, 2001). Supervisor. Subjective	C	0.47 *	50 teams. Two organizations. Korea	n/a
7. Meneghel, Salanova & Martínez (2016) [77]	Examine the relationship between collective positive emotions at work and team resilience	Broaden-and-build theory of positive emotions (Fredrickson, 1998, 2001)	HP	n/a	Five collective emotions: enthusiasm, optimism, satisfaction, comfort, and relaxation. Faces Scale (Kunin 1955). Last year	Employees; Team	Team performance scale: In-role (n/a; adaptation from Godman and Svyantek (1999). Supervisor. Subjective	C	0.15 *	216 teams. Commercially oriented service organizations (shops, bars, restaurants and physiotherapists’offices). Spain	4,99
							Team performance scale: Extra-role (n/a; adaptation from Godman and Svyantek (1999). Supervisor. Subjective		0.20 *		
8. Peñalver, Salanova, Martínez & Schaufeli (2017) [81]	Examine the mediating role of group social resources between group positive affect and group performance	Broaden-and-build theory of positive emotions (Fredrickson, 1998)	HP	The affective composition of the group members (Barsade & Gibsonm 1998), resulting from feeling similar levels of individual emotions when people work together (Barsade, Knight, 2015)	Group positive affect (4-items; Salanova, Llorens, Cifre, & Martínez, 2012). Past year. At work	Employees: Individual	Team performance scale: In-role (3-items; adaptation of the scale of Goodman & Svyantek, 1999). Supervisor. Subjective	C	0.13 **	417 teams. Different companies. Spain	5,14
							Team performance scale: Extra-role (3-items; adaptation of the scale of Goodman & Svyantek, 1999). Supervisor. Subjective		0.14 **		
9. Rego, Júnior, Pina, Stallbaum & Neves (2016) [38]	Examine whether (1) store positive affective tone predicts store performance through creativity, and (2) store negative affective tone enhances the relationship between positive affective tone and creativity	Emotional contagion (Hatfield et al., 1994); Mood-as-information theory (Forgas & Vargas, 2000); Broaden-and-build theory (Fredrickson, 2001)	HP	Group (store’s) affective tone is defined as consistent or homogeneous affective reactions within a group (George, 1990)	Positive affective tone (3-items; Turban et al., 2009). 6 months	Supervisors; Team	Store creativity (13-items; Zhou & George, 2001). Supervisor. Subjective	C	0.46 ***	94 stores’ supervisors. Retail organization (appliances sector). Brazil	12,5
							Sales achievement. Top management. Objective	CL	performance in the semestre (0.25 **); performance subsequent semester (0.07ns)		
10. Seong & Choi (2014) [78]	Examine the relationship between positive affect of leaders and members with group-level fit perceptions and subsequent group processes and performance	Affective-consistency perspective (Yu, 2009); Broaden-and-build theory (Fredrickson, 1998)	HP	n/a	Delighted; pleased; happy; comfortable; satisfied; relaxed (6-items; Posner, Russell, & Peterson, 2005). In general. At work	Team members; Team	Team performance scale (4- items; adaptation of the scale of Zellmer-Bruhn & Gibson, 2006). Supervisors. Subjective	C	0.02ns	96 teams. Defense industry. Korea	10,35
11. Shin (2014) [79]	Explore group-level mechanisms linking positive group affective tone and team creativity	Group creativity model (Paulus & Dzindolet, 2008); Broaden-and-build theory (Fredrickson, 1998, 2001)	HP	Group affective tone is defined as consistent or homogeneous affective reactions within a group (George, 1990)	PANAS (4-items; Watson et al., 1988). One week frame	Team members; Individual	Creativity scale (5-items; Zhou & George, 2001) Supervisors. Subjective	C	0.40 ***	98 teams. Different companies (banking and finance, service, and manufacturing). Korea	5,8
12. Tanghe, Wisse & Van der Flier (2010) [46]	Examine whether positive group affective tone is positively associated with team effectiveness and if this effect is stronger for higher levels of group identification	Broaden-and-build theory (Fredrickson, 1998); Social identity perspective (Turner, 1985); Circumplex model of affect (Larsen & Diener, 1992)	HP	Group affective states refers to the shared emotions or shared moods (George, 2002)	Positive affect scale (6-items; Larsen & Diener, 1992). Felt at very particular moment	Employees; Individual	Willingness to engage in OCB (5-items; Moorman and Blakely, 1995) Employees. Subjective	C	0.40 **	71 teams. Service organizations. n/a	2-4
							Perceived team performance (4-items; n/a). Employees. Subjective		0.19ns		
13. Tsai, Chi, Grandey & Fung (2012) [85]	Explore boundary conditions of the relationship between positive group affective tone and team creativity	Group-centrism perspective (Kruglanski et al., 2006); ’Dual-tuning’ perspective (Kruglanski, Pierro, Mannetti, & De Grada, 2006)	HP	Group affective tone is defined as consistent or homogeneous affective reactions within a group (George, 1990)	PANAS (10-items; Watson et al., 1988). Past week. Team meetings	Employees; Individual	Team creativity scale (3-items; Van der Vegt & Janssen, 2003). Supervisors. Subjective	C	0.09 ns	68 teams. High-technology firms. Taiwan	5,9
14. Tu (2009) [83]	Examine how contextual factors moderate the relationship between team affective tone and team creativity	Mood-as-input model (Martin & Stoner, 1996)	HP	n/a	PANAS (10-items; Watson et al., 1988). Past week. At work	Employees; Individual	Team creativity. Adaptation of team creativity scales (Scott & Bruce, 1994; Zhou & George, 2001). Employees and supervisors. Subjective	C	0.34 *	106 teams. Different industries (computer; semi-conductors; audio and video electronic). Taiwan	5,71

^a^ HP = Happy-Productive; PH = Productive-Happy; ^b^ GA = Group Affect; PANAS = Positive Affect and Negative Affect Schedule; ^c^ P = Performance; ^d^ C = Cross-sectional design; TL = Time-lagged design; ^e^ R = correlation coefficient; ns = not significant; * = *p* < 0.05; ** = *p* < 0.01; *** = *p* < 0.001.

**Table A3:** Description of the studies on Team engagement – Collective Performance.

Source	Study Goal	Theories	HP ^a^	EG Definition	EG Measure ^b^	EG Informant EG Referent	P Measure ^c^	Design ^d^	R ^e^	Sample	Team Size
1. Costa, Passos, & Bakker (2015) [87]	Understand whether the two types of conflict impact differently on proximal (team work engagement) and distal (team performance) team outcomes, directly; simultaneously, to explore the moderator influence of team conflict on the job demands-resources model	Job demands-resources model (Demerouti & Bakker, 2011); Broaden-and-build theory (Fredrickson, 1998)	HP	Team work engagement is defined as a shared, positive and fulfilling, motivational emergent state of work-related well-being (Costa et al., 2014)	Team work engagement scale (9-items; Costa et al., 2014)	Team members; Team	Number of publications, oral presentations in congresses; leader; objective	C	0.24 *	82 research teams. Southern European country	3.41
2. Cruz-Ortiz, Salanova, & Martínez (2013) [57]	Test the relationship between transformational leadership, team work engagement and team performance	Healthy and resilient organizations model (Salanova, 2008)	HP	Work engagement is defined as a positive, fulfilling, work-related state of mind that is characterized by vigour, dedication, and absorption (Schaufeli et al., 2002)	UWES (18-items; Schaufeli et al., 2002; Salanova et al., 2003)	Employees; Team	In-role; 3-items; employees; subjective	C	0.37 **	58 teams different SMEs. Spain	8.94
							Extra-role; 3-items; employees; subjective		0.38 **		
3. García-Buades, Martínez-Tur, Ortiz-Bonnín, & Peiró (2016) [90]	Examine the moderating role of team climate for innovation on the relationship between team engagement and service performance	Job demands-resources model of work engagement (Bakker & Demerouti, 2008)	HP	Team engagement is defined as ’a positive, fulfilling, work-related and shared psychological state characterized by team work vigor, dedication and absorption which emerges from the interaction and shared experiences of the members of a work team’ (Torrente, Salanova, Llorens, & Schaufeli, 2012)	UWES-9 spanish version (9-items; Schaufeli, Bakker, & Salanova, 2006)	Employees; Individual	Service quality: functional and relational (22-items; customers; subjective)	C	0.18ns; 0.26 *	86 reception and restaurant teams. Spanish mediterranean coast	4
							Satisfaction and loyalty (6-items; customers; subjective)		0.07ns; 0.09ns		
4. Luu (2017) [92]	Investigate the relationship between collective job crafting and team service recovery performance via the mediation mechanism of team work engagement	Attitude theory (Bagozzi, 1992)	HP	Work engagement is a positive, fulfilling, work-related and shared psychological state of mind (Salanova et al., 2003)	UWES (3-items; Tims et al., 2013)	Employees; Team	Service recovery performance; 5-items; leaders; subjective	C	0.39 ***	181 clinicians teams. Vietnam	7.23
5. Makikangas, Aunola, Seppala, & Hakanen (2016) [88]	Examine if individual and team work engagement are associated with team members’ perceived team performance	Job demands-resources model of work engagement (Bakker & Demerouti, 2007)	HP	Work engagement is a positive, fulfilling, and rather consistent state of mind characterized by vigour, dedication, and absorption (Schaufeli et al., 2002).	Team Work Engagement Scale (4-items; Costa et al., 2014)	Employees; Team	Perceived team performance; 5-items; employees; subjective	C	0.30 **	102 teachers and administrative teams. Finnish	10.53
6. McClelland, Leach, Clegg, & McGowan (2014) [93]	Examine the antecedents and outcomes of team-level collaborative crafting	Role adjustments lead to improve performance through changes in job content, higher self-efficacy, and higher motivation (Clegg & Spencer, 2007)	HP	Work engagement is a job holder’s affective psychological connection to his/her work tasks (Schaufeli & Bakker, 2004)	UWES (9-items; Schaufeli et al., 2006)	Employees; Individual	Team achievements, efficiency, work quality, and mission fulfilment; 4-items; supervisors; subjective	C	0.30 **	242 retaliers and insurance provider call centre teams. United Kingdom	11.1
7. Salanova, Agut, & Peiró (2005) [91]	Examine the mediating role of service climate in the prediction of employee performance and customer loyalty	Job demands-resources model of work engagement (Bakker & Demerouti, 2008)	HP	Engagement is a positive, fulfilling, work-related state of mind that is characterized by vigor, dedication, and absorption (Shaufeli et al., 2002)	Work Engagement Scale (Schaufeli et al., 2002)	Employees; Individual	Empathy and excellent job performance; 6-items; customers; subjective	C	0.10ns	114 reception and restaurant units. n/a	3
8. Tims, Bakker, Derks, & Rhenen (2013) [62]	Hypothesize that team job crafting relates positively to team performance through team work engagement	Job demands-resources model (Bakker & Demerouti, 2007); Broaden-and-build theory (Fredrickson, 1998)	HP	Work engagement is defined as a positive, fulfilling, work-related state of mind that is characterized by vigor, dedication, and absorption (Schaufeli et al., 2002)	UWES (9-items/3-items; Schaufeli et al., 2006)	Employees; Individual/team	Williams and Anderson (1991).5-items; employees;subjective	C	0.54 **	54 health services teams company. Netherlands	16.12
9. Torrente, Salanova, Llorens, & Schaufeli (2012) [58]	Analyze the mediating role of team work engagement between team social resources, and team performance	Job demands-resources model (Demerouti et al., 2001)	HP	Work engagement is a positive, fulfilling, work-related and shared psychological state characterized by teams work vigor, dedication and absorption which emerges from the interaction and shared experiences of the members of a work team (Salanova et al., 2003)	Team work engagement scale (9-items; Torrente, Salanova, Llorens, & Schaufeli, 2013)	Employees; Team	In-role; 3-items; supervisors; subjective	C	0.25 *	62 teams from 13 enterprises. n/a	n/a
							Extra-role; 3-items; supervisors; subjective		0.12ns		

^a^ HP = Happy-Productive; PH = Productive-Happy; ^b^ EG = Engagement; UWES = Ultrecht Work Engagement Scale; ^c^ P = Performance; ^d^ C = Cross-sectional design; CL = Cross-lagged design; ^e^ R = correlation coefficient; ns = not significant; * = *p* < 0.05; ** = *p* < 0.01; *** = *p* < 0.001; Sources 3, 8, & 10 r = mean correlation of vigour, dedication, and absorption.
