# Supplementary material for: Happy-Productive Teams and Work Units: A Systematic Review of the ‘Happy-Productive Worker Thesis’
Source: Int J Environ Res Public Health. 2019 Dec 20;17(1):69. doi: 10.3390/ijerph17010069 (PMC6981377; doi:10.3390/ijerph17010069)
Supplement: Supplementary file 1 [file ijerph-17-00069-s001.pdf]

Systematic search – PsycINFO and PsycARTICLES

SATISFACTION:

KW satisfaction AND KW (performance or productiv* or efficien* or effective* or "customer satisfaction" or "organizational citizenship behavior" or innov* or creativ*) AND AB ((team* or "work unit" or "work-unit" or "unit" or "units" or "workgroup" or "work group") AND AB (empirical or data or sample or review or metanalysis or meta-analysis) NOT AB (students or athletes or sport or adolescents or children or disorder)

GROUP AFFECT:

AB ("group affect*" or "positive affect" or "group positive affect" or "positive emotions" or "team mood" or "group mood" or "positive mood") AND AB (performance or productiv* or efficien* or effective* or "customer satisfaction" or "organizational citizenship behavior" or innov* or creativ*) AND AB (team* or "work unit" or "work-unit" or "unit-level" or "units" or "workgroup" or "work group") AND AB (empirical or data or sample or review or metanalysis or meta-analysis) NOT AB (students or athletes or sport or adolescents or children or disorder)

ENGAGEMENT:

KW engagement AND KW (performance or productiv* or efficien* or effective* or "customer satisfaction" or "organizational citizenship behavior" or innov* or creativ*) AND AB (team* or "work unit" or "work-unit" or "unit-level" or "units" or "workgroup" or "work group") AND AB (empirical or data or sample or review or metanalysis or meta-analysis) NOT AB (students or athletes or sport or adolescents or children or disorder)
